# Supplementary material for: Adjusting for principal components can induce collider bias in genome-wide association studies
Source: PLoS Genet. 2024 Dec 16;20(12):e1011242. doi: 10.1371/journal.pgen.1011242 (PMC11684764; doi:10.1371/journal.pgen.1011242)
Supplement: S1 Text — Includes further details and investigation of principal component behavior in WHI SHARe and TOPMed samples, proofs and additional simulation studies validating the theoretical results presented in the main paper, and a list of WHI investigators. (PDF) [file pgen.1011242.s001.pdf]

Supplemental methods and results: Adjusting for principal components can induce collider bias in genome-wide association studies

Kelsey E. Grinde* Brian L. Browning Alexander P. Reiner

Timothy A. Thornton Sharon R. Browning

*kgrinde@macalester.edu

Contents

List of Supplemental Figures **3**

List of Supplemental Tables **5**

A Additional TOPMed PC-Genotype Correlation Plots **6**

B Comparison of PCA Pre-Processing Choices **9**

B.1 WHI SHARe 9

B.1.1 Proportion of Variance Explained 9

B.1.2 Correlation between PCs and Genotypes 10

B.2 TOPMed 15

B.2.1 Correlation between PCs and Genotypes 15

B.2.2 Proportion of Variance Explained 22

C Investigation of PCs in a European American Population **29**

D Comparison of PCs and Model-Based Admixture Proportions **34**

E GWAS Effect Size Derivations **37**

E.1 Assumed data-generating mechanism 37

E.2 Expected effect size estimates 39

E.2.1 Unadjusted model 40

E.2.2 Admixture proportion adjusted model 42

E.2.3 Principal component adjusted model 42

E.3 Simulations validating theory 44

F Spurious Association Simulation Studies **49**

F.1 WHI SHARe 49

F.2 TOPMed 53

G List of WHI Investigators **57**

Supplemental References **58**

List of Supplemental Figures

A.1 Correlation between PCs 5–20 and genotypes in JHS African Americans . . . 7

A.2 Correlation between PCs 5–20 and genotypes in COPDGene African Americans 8

B.1 Correlation between PCs and genotypes using different LD pruning thresholds. 12

B.2 Correlation between PCs and genotypes using different LD pruning windows. 13

B.3 Correlation between PCs and genotypes using a data-based filtering process. 14

B.4 Correlation between PCs and genotypes in JHS using LD pruning with 0.5 Mb windows. 16

B.5 Correlation between PCs and genotypes in JHS using LD pruning with 10 Mb windows. 17

B.6 Correlation between PCs and genotypes in COPDGene using LD pruning with 0.5 Mb windows. 18

B.7 Correlation between PCs and genotypes in COPDGene using LD pruning with 10 Mb windows. 19

B.8 Correlation between PCs and genotypes in COPDGene using LD pruning with $r^2 < 0.05$. 20

B.9 Correlation between PCs and genotypes in COPDGene using LD pruning with a stricter MAF filter. 21

B.10 Scree plot for PCs generated without any LD-based pruning or filtering in JHS. 22

B.11 Scree plot for PCs generated after LD pruning with an r^2 threshold of 0.1 and window size of 0.5 Mb in JHS. 23

B.12 Scree plot for PCs generated after LD pruning with an r^2 threshold of 0.1 and window size of 10 Mb in JHS. 24

B.13 Scree plot for PCs generated without any LD-based pruning or filtering in COPDGene African Americans. 25

B.14 Scree plot for PCs generated after LD pruning with an r^2 threshold of 0.1 and window size of 0.5 Mb in COPDGene. 26

B.15 Scree plot for PCs generated after LD pruning with an r^2 threshold of 0.1 and window size of 10 Mb in COPDGene. 27
B.16 Scree plot for PCs generated after LD pruning with an r^2 threshold of 0.1 and window size of 0.5 Mb in COPDGene. 28
C.1 SNP loadings in COPDGene European Americans. 31
C.2 SNP loadings in COPDGene European Americans after excluding known high LD regions. 32
C.3 SNP loadings in COPDGene European Americans after LD pruning. 33
D.1 Scatterplots of estimated admixture proportions versus the first four PCs, without LD-based filtering or pruning. 35
D.2 Scatterplots of estimated admixture proportions versus the first four PCs, with LD-based filtering and pruning. 36
E.1 Barplot of simulated admixture proportions. 45
E.2 Observed versus expected and true effect sizes from unadjusted GWAS models. 46
E.3 Observed versus expected and true effect sizes from admixture proportion adjusted GWAS models. 47
E.4 Observed versus expected and true effect sizes from principal component adjusted GWAS models. 48
F.1 WHI SHARe simulation results. 50
F.2 Manhattan plots from GWAS in WHI SHARe using either LD pruning or filtering, but not both. 52
F.3 Correlation between fake PCs and genotypes in TOPMed JHS. 54
F.4 Manhattan plots for GWAS models adjusting for fake PCs in TOPMed JHS. 55
F.5 QQ plots for GWAS models adjusting for fake PCs in TOPMed JHS. 56

List of Supplemental Tables

B.1 Percent of variance explained by each PC in WHI SHARe. 10

A Additional TOPMed PC-Genotype Correlation Plots

In Fig 2 we present PC-genotype correlation plots for JHS and COPDGene African Americans when PCs were generated without any prior LD-based filtering or exclusions. The figure focuses on just the top four PCs. Here, we present additional results for PCs 5–20. Fig A.1 presents results for JHS and Fig A.2 for COPDgene.

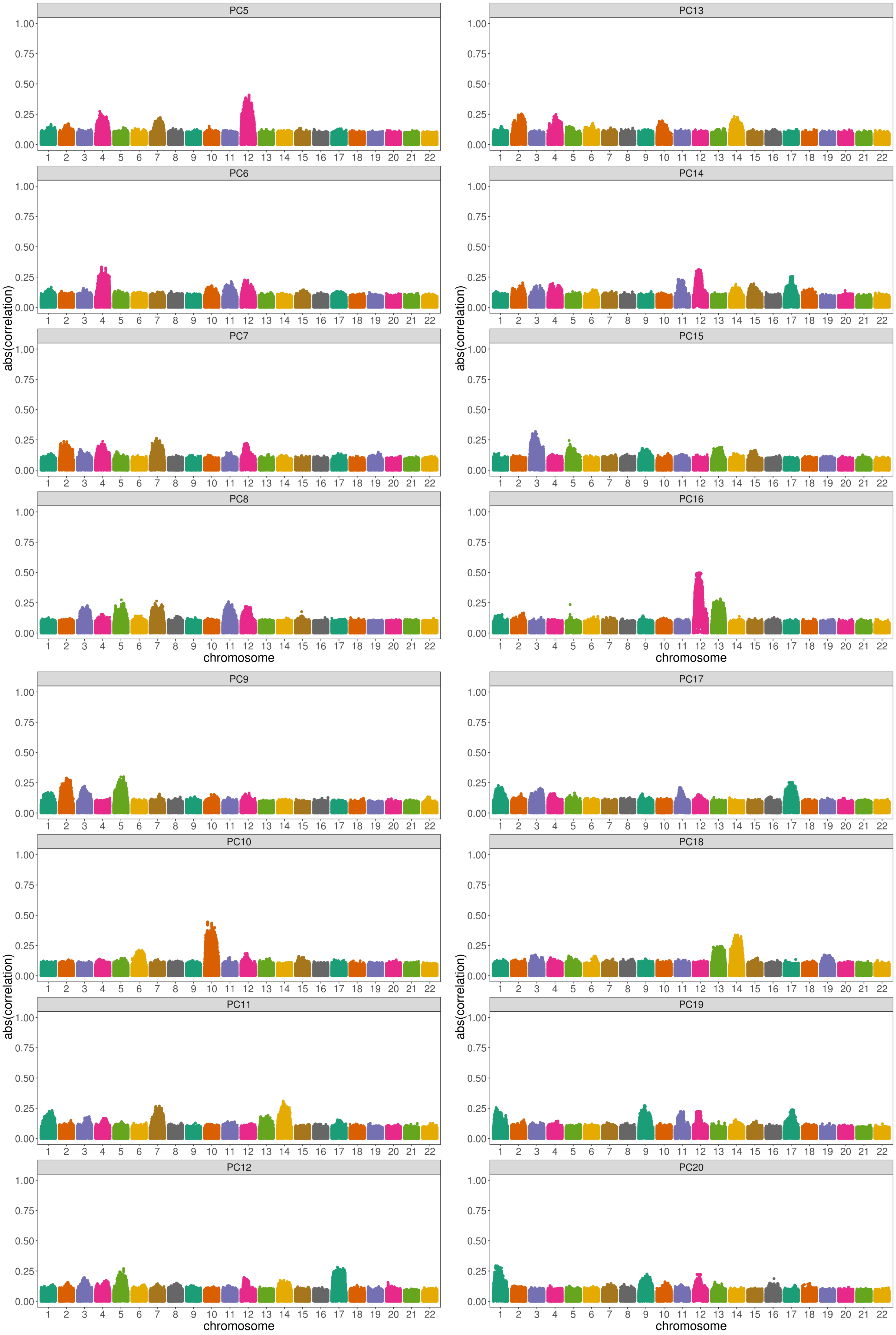

Fig A.1. Correlation between PCs 5–20 and genotypes in JHS African Americans when PCs were generated without any prior LD-based filtering or exclusions.

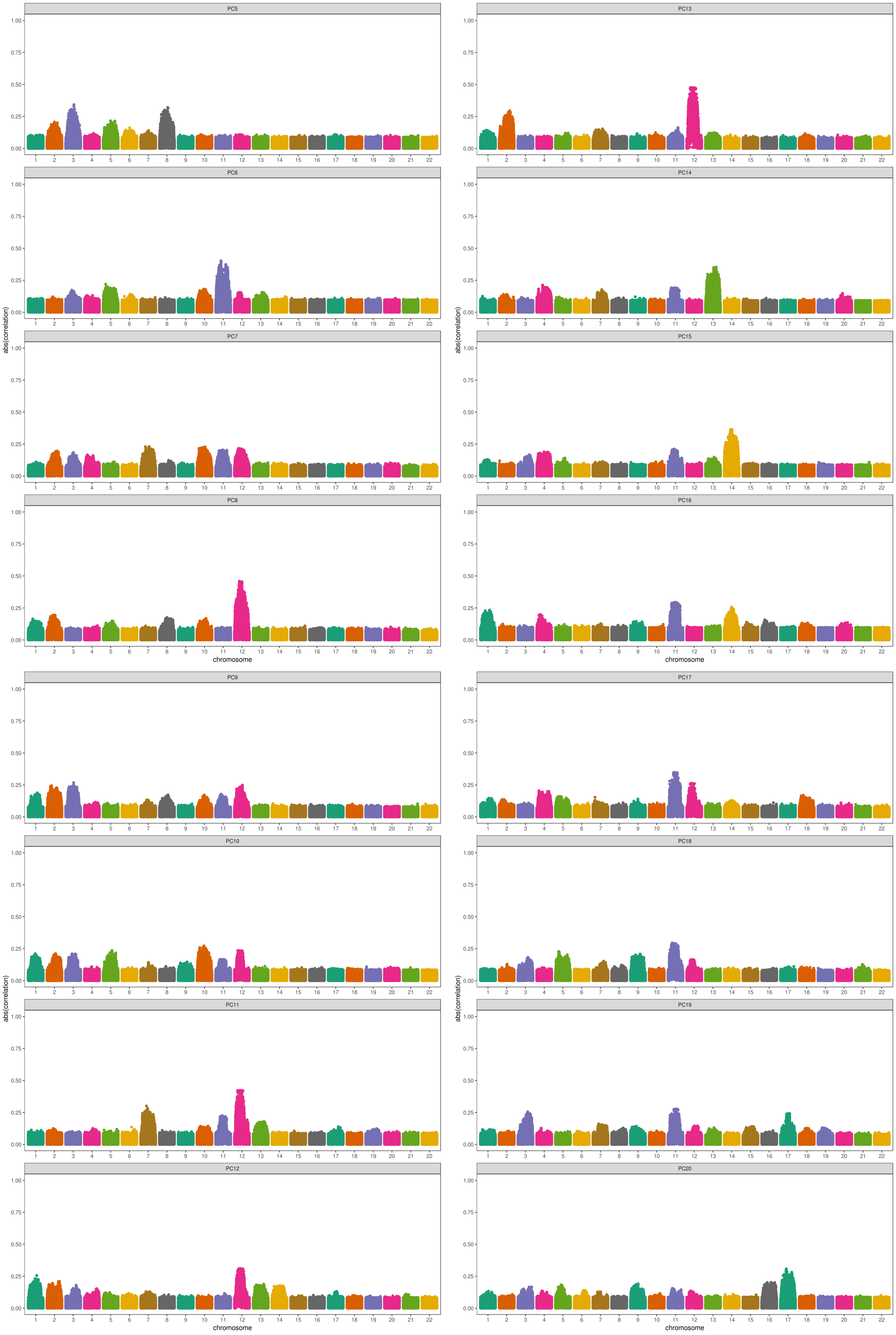

Fig A.2. Correlation between PCs 5–20 and genotypes in COPDGene African Americans when PCs were generated without any prior LD-based filtering or exclusions.

B Comparison of PCA Pre-Processing Choices

B.1 WHI SHARe

In the main paper, we show that adjusting for principal components that capture small regions of the genome rather than genome-wide ancestry can induce spurious associations in genome-wide association studies. This problematic behavior occurred in our analysis of genotype data from WHI SHARe African Americans when PCs were generated using all 551,025 available SNPs, and when we excluded regions identified in the literature as being potentially problematic for PCA (Table 1). However, problems were ameliorated when we used PCs that were generated after strict LD pruning, using an r^2 threshold of 0.1 and window size of 0.5 Mb.

B.1.1 Proportion of Variance Explained

Results in the main paper focus on four sets of PCs:

- *none*: PCs generated without any prior LD-based pruning or filtering
- *exclude*: PCs generated after excluding regions in Table 1, but no LD pruning
- *prune*: PCs generated after LD pruning with an r^2 threshold of 0.1 and window size of 0.5 Mb
- *both*: PCs generated after both LD pruning and excluding regions from Table 1

In Table B.1 we present the proportion of variance explained by each of these PCs. Across all pre-processing choices, the first PC explains a considerably higher proportion of variance than all others.

Table B.1. Percent of variance explained by each PC in WHI SHARe.
Columns correspond to the four different pre-processing choices described above and presented in Fig 3.

	none	exclude	prune	both
PC1	1.477	1.477	0.694	0.691
PC2	0.083	0.081	0.093	0.093
PC3	0.081	0.080	0.058	0.058
PC4	0.079	0.078	0.048	0.048

B.1.2 Correlation between PCs and Genotypes

In this section, we further investigate the behavior of PCs generated after different filtering techniques.

Many authors have suggested using an r^2 threshold of 0.2 for LD pruning prior to running PCA[1,2,3,4,5,6,7,8]. Furthermore, this threshold is the default for LD pruning software such as `SNPRelate`[9]. However, in our analysis of WHI SHARe data, we found that using an r^2 threshold of 0.2 prior to running PCA still led to one of the top PCs (the fourth) being highly correlated with small regions of the genome, while if we used a stricter threshold of 0.1 the peaks have disappeared (at least for the top four PCs). See Fig B.1 for a comparison of the correlation between PCs and genotypes in WHI SHARe African Americans across different choices of r^2 threshold.

When performing LD pruning, another choice that practitioners have to make is the window size. In the literature, various window sizes have been suggested, including 10 Mb[10], 2 Mb[2], or 0.5 Mb (the `SNPRelate` default), and others have suggested that window size may not have a big impact[4]. Similar to the latter, in our analysis of WHI SHARe data we see little difference in the correlation between PCs and genotypes across different choices of window sizes: see Fig B.2. Smaller window sizes are less computationally intensive, so we used the window size of 0.5 Mb for the remainder of our analyses.

Finally, we also considered filtering out regions that were highly correlated with PCs in our own data, as has been done previously[7,1]. To implement this data-based filtering, we investigated the SNP loadings for each of the top four PCs. Starting with the second PC, we

found the SNP on each chromosome with the largest loading: if this loading was larger than 0.005, we excluded the SNP and all SNPS within M Mb; if the loading was small, we kept all SNPs on the chromosome. (We considered $M = 1, 5, 10$, and 20 Mb.) We repeated this process for PCs 3 and 4, then re-ran PCA using the remaining SNPs. Using these new PCs, we re-calculated SNP loadings and looked to see if there were still regions of the genome that were driving the PCs. If so, we repeated the entire process. This data-based filtering process is very tedious, and even after four rounds of exclusions with $M = 5$ Mb we found that the problematic behavior did not totally go away (Fig B.3).

In WHI SHARe data, at least, strict LD pruning is the most effective of the pre-processing steps that we considered in eliminating the correlation between PCs and genotypes in small regions of the genome.

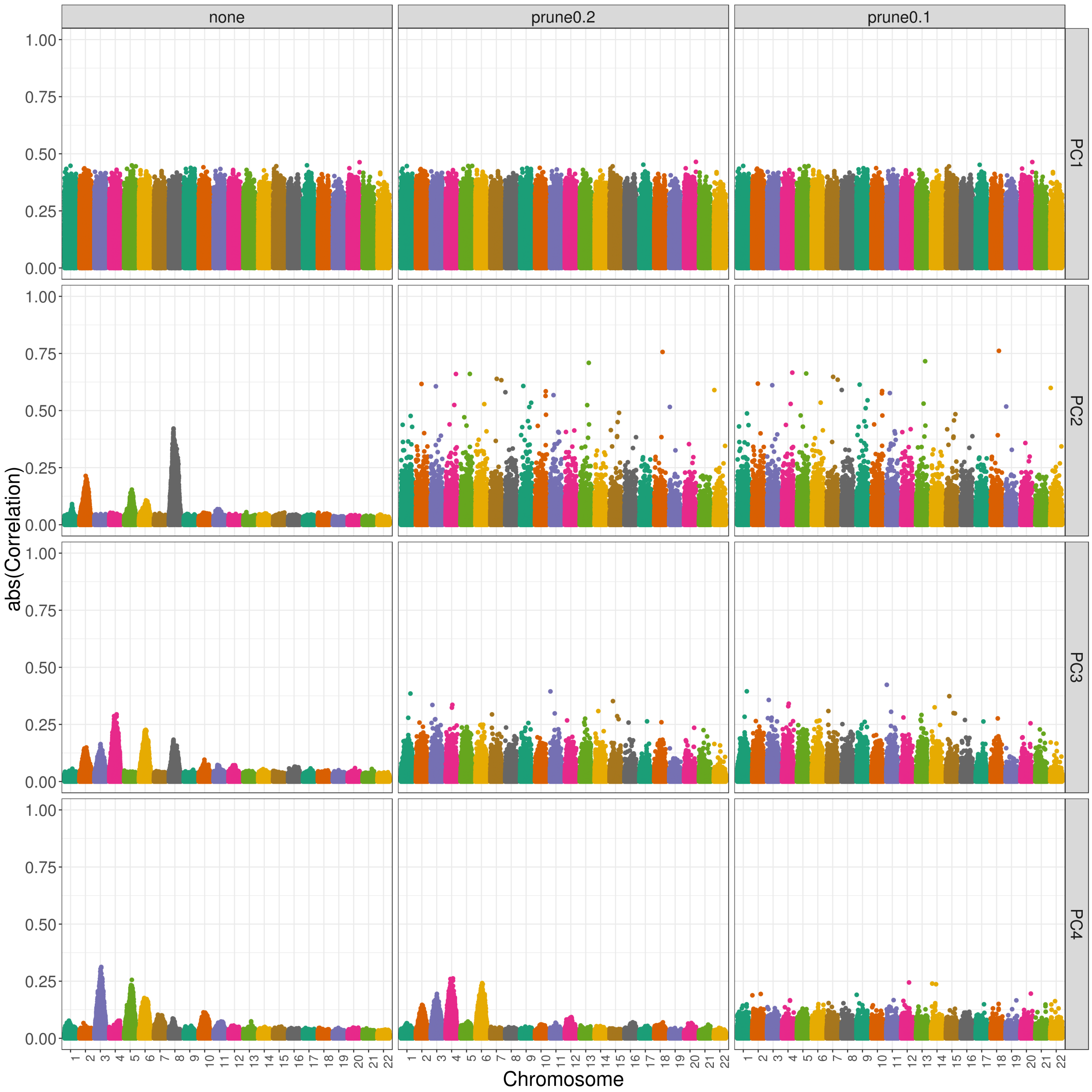

Fig B.1. Correlation between PCs and genotypes in WHI SHARe African Americans using different LD pruning thresholds.

Each panel plots the absolute value (abs) of the correlation between principal components and genotypes on the y-axis versus the position along the genome on the x-axis. Panels are organized vertically according to which PC is being investigated (1, 2, 3, 4) and horizontally according to what r^2 threshold was used when running LD pruning prior to PCA (*none*: no LD pruning, *prune0.2*: LD pruning with an r^2 threshold of 0.2 and window size of 0.5 Mb, and *prune0.1*: LD pruning with an r^2 threshold of 0.1 and window size of 0.5 Mb).

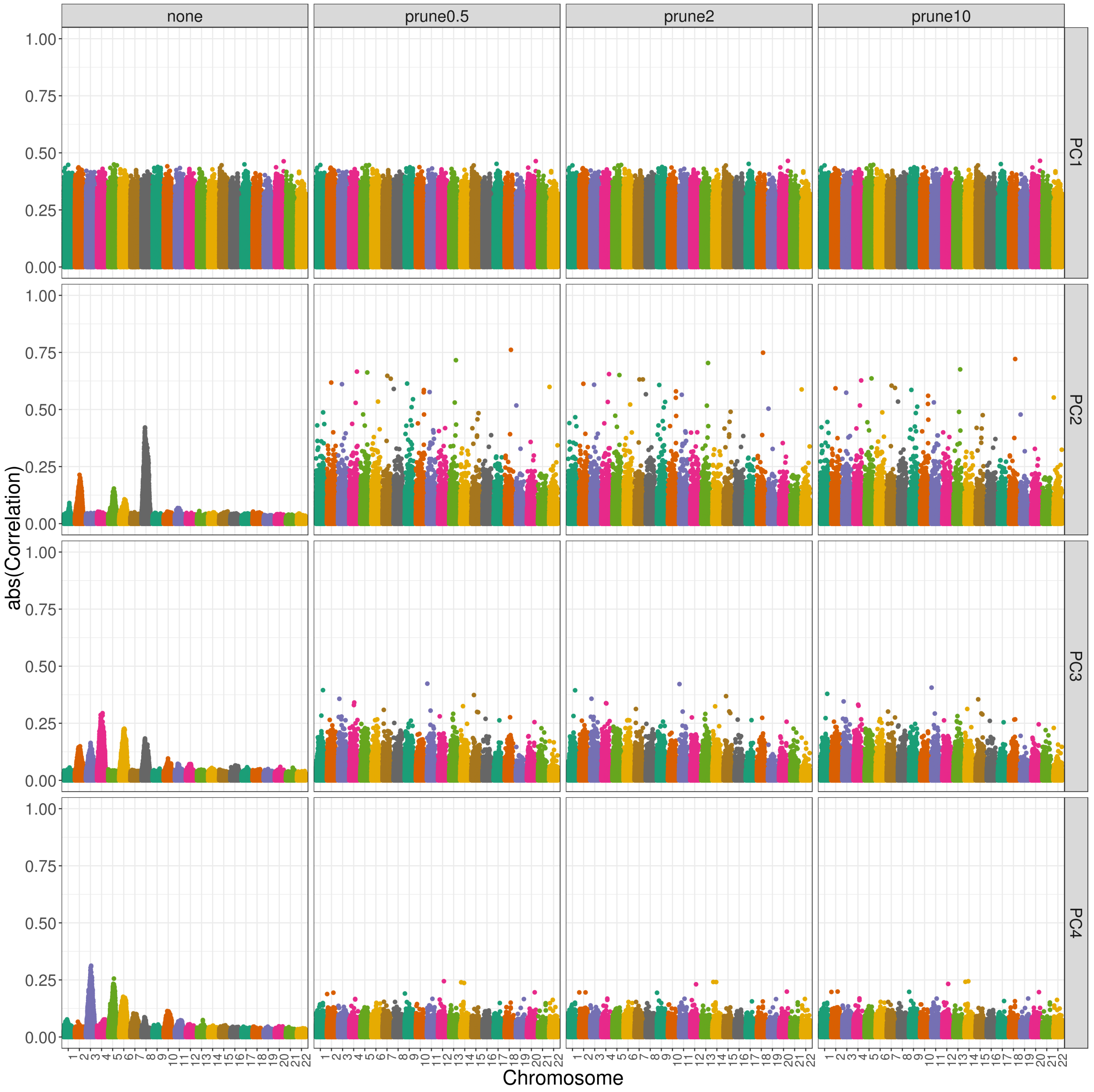

Fig B.2. Correlation between PCs and genotypes in WHI SHARe African Americans using different LD pruning window sizes.

Each panel plots the absolute value (abs) of the correlation between principal components and genotypes on the y-axis versus the position along the genome on the x-axis. Panels are organized vertically according to which PC is being investigated (1, 2, 3, 4) and horizontally according to what window size was used when running LD pruning prior to PCA (*none*: no LD pruning, *prune0.5*: LD pruning with an r^2 threshold of 0.1 and window size of 0.5 Mb, *prune2*: LD pruning with an r^2 threshold of 0.1 and window size of 2 Mb, and *prune10*: LD pruning with an r^2 threshold of 0.1 and window size of 10 Mb).

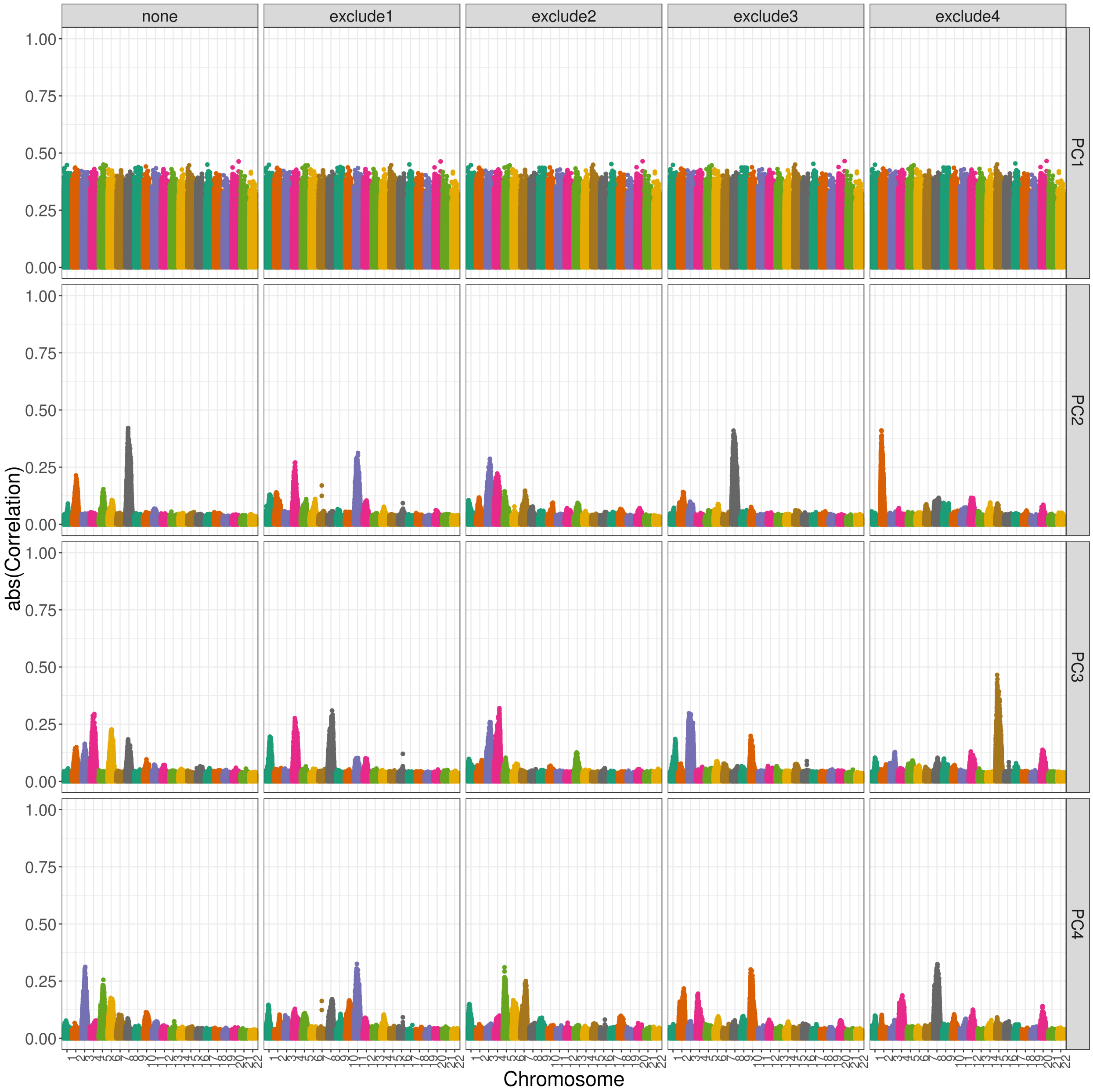

Fig B.3. Correlation between PCs and genotypes in WHI SHARe African Americans after multiple rounds of data-based exclusions.

Each panel plots the absolute value (abs) of the correlation between principal components and genotypes on the y-axis versus the position along the genome on the x-axis. Panels are organized vertically according to which PC is being investigated (1, 2, 3, 4) and horizontally according to the number of iterations of our procedure for excluding regions highly correlated with PCs in this sample (*none*: no exclusions, *exclude1*: one round of exclusions, *exclude2*: two rounds of exclusions, etc.).

B.2 TOPMed

B.2.1 Correlation between PCs and Genotypes

In WHI SHARe African Americans, performing LD pruning with an r^2 threshold of 0.1 and window size of 0.5 Mb produces PCs that do not capture local genomic features. Using these same parameters in the TOPMed Jackson Heart Study sample (Fig B.4) does show some improvement relative to performing PCA without any prior LD-based exclusions or pruning (Fig 2), but we still see some peaks in the PC-genotype correlation plots, particularly in the case of the fourth PC. After increasing the window size to 10 Mb (Fig B.5), the peaks disappear.

In TOPMed COPDGene African Americans, however, we continue to see peaks in the PC-genotype correlation plots with both a window size of 0.5 Mb (Fig B.6) and the wider window size of 10 Mb (Fig B.7). Dropping the r^2 threshold did not solve the issue either (Fig B.8). We also explored the impact of using a more stringent MAF filter (Fig B.9): although the patterns change slightly, the overarching issue of PCs capturing local genomic features is not resolved.

Our results indicate that the optimal choice of LD pruning parameters may be dataset-specific.

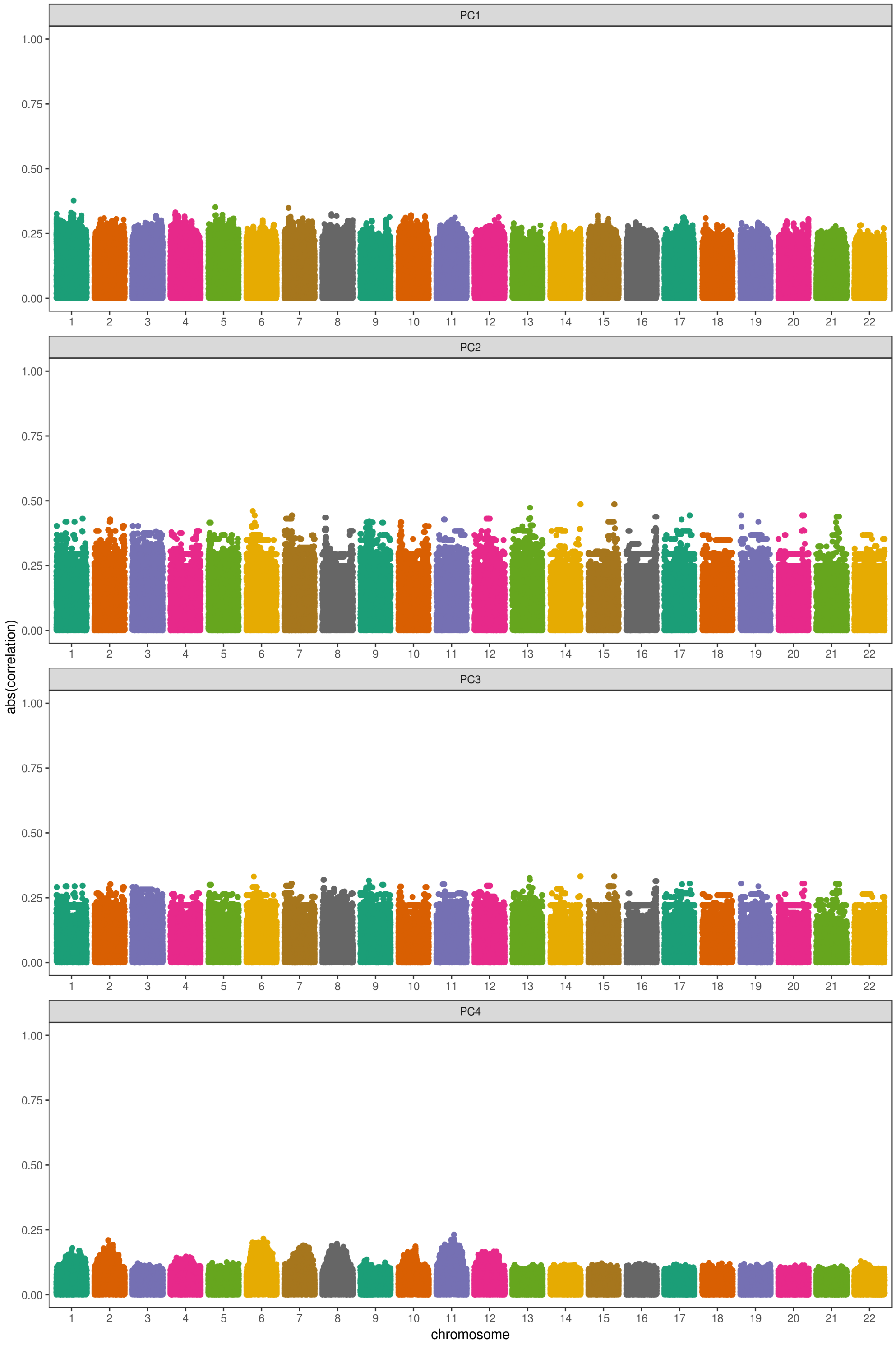

Fig B.4. Correlation between PCs and genotypes in JHS African Americans using LD pruning with an r^2 threshold of 0.1 and window size of 0.5 Mb.
Each panel plots the absolute value (abs) of the correlation between principal components and genotypes on the y-axis versus the position along the genome on the x-axis. Panels are organized vertically according to which PC is being investigated (1, 2, 3, 4).

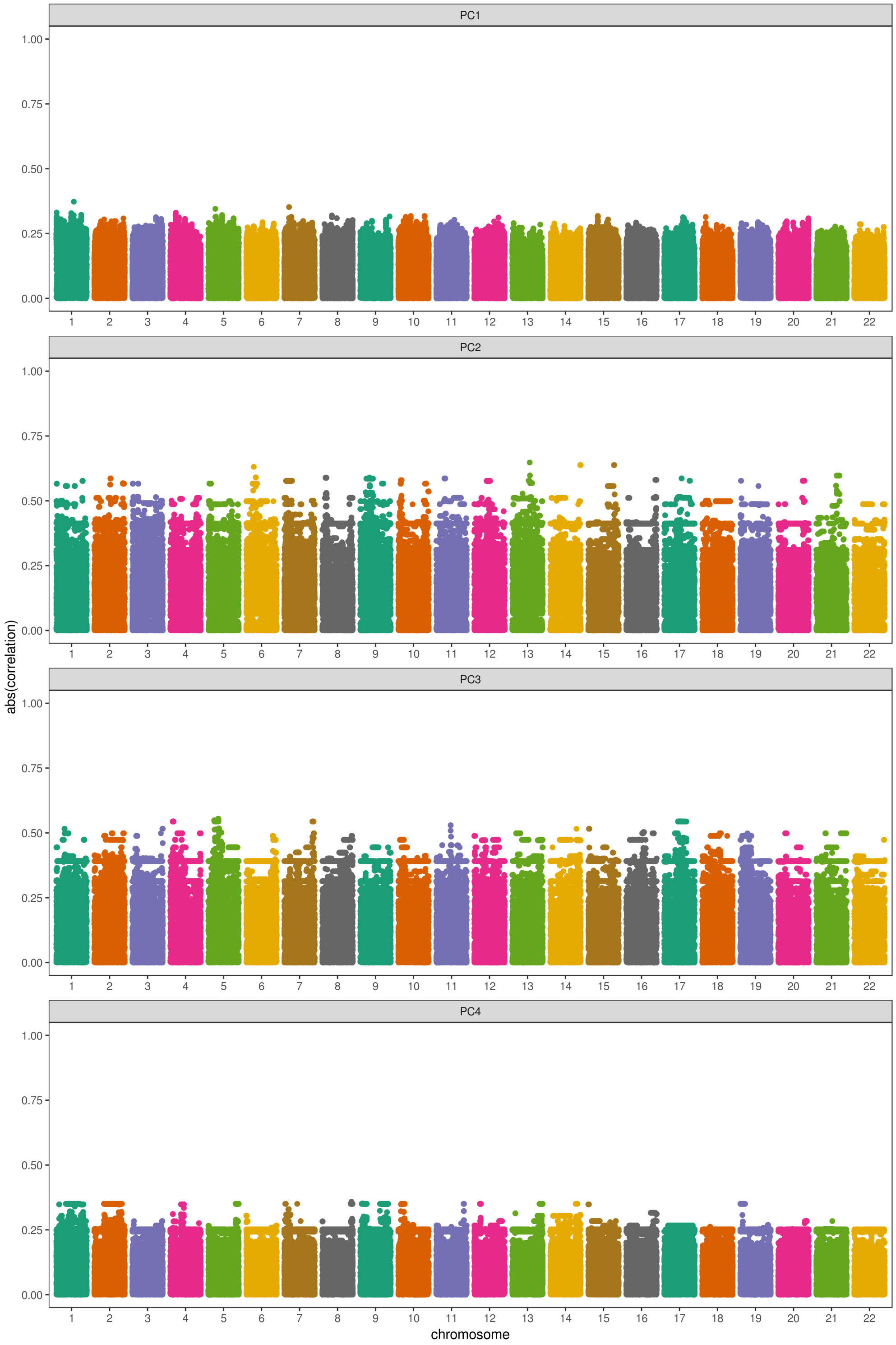

Fig B.5. Correlation between PCs and genotypes in JHS African Americans using LD pruning with an r^2 threshold of 0.1 and window size of 10 Mb.

Each panel plots the absolute value (abs) of the correlation between principal components and genotypes on the y-axis versus the position along the genome on the x-axis. Panels are organized vertically according to which PC is being investigated (1, 2, 3, 4).

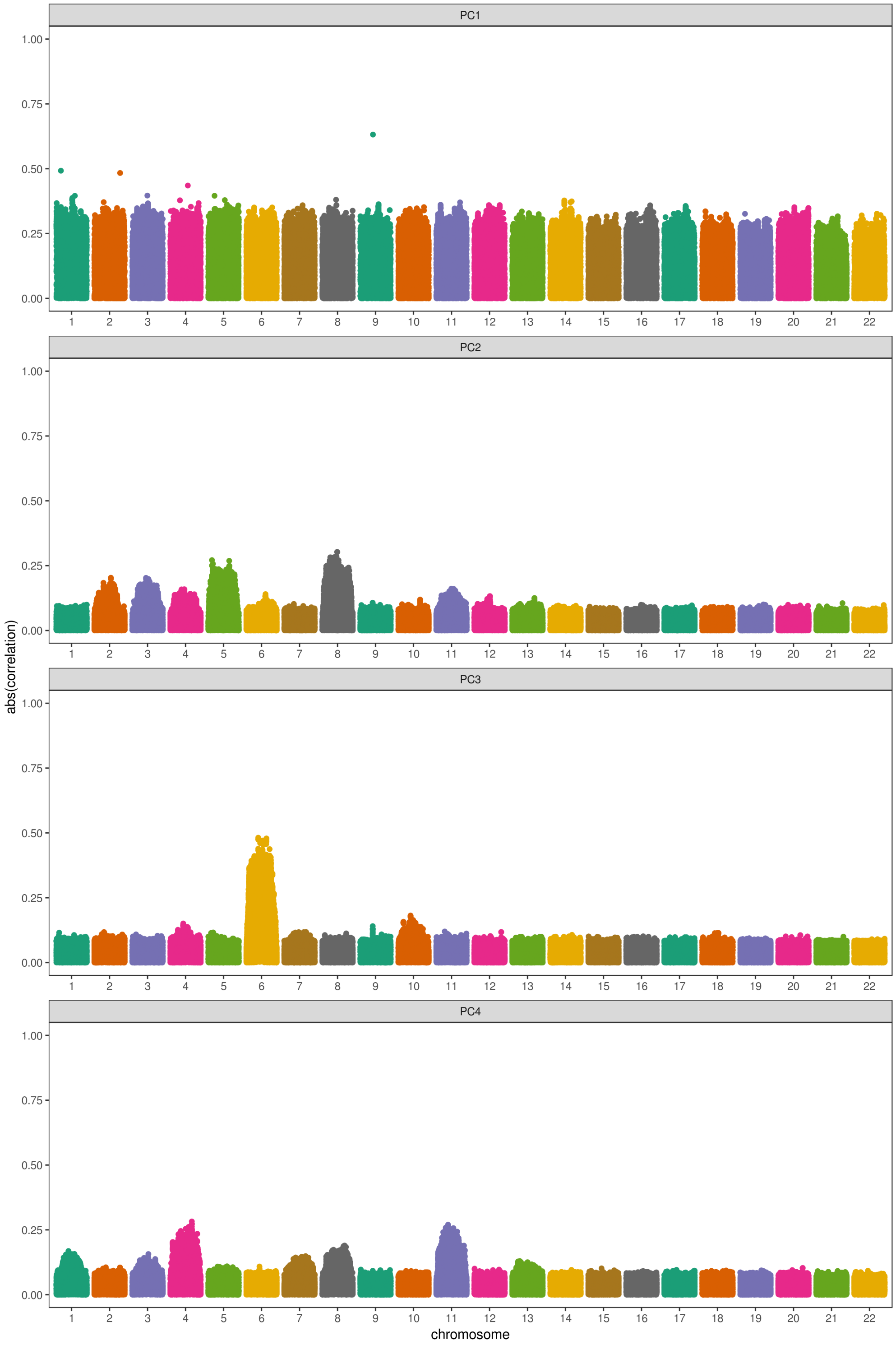

Fig B.6. Correlation between PCs and genotypes in COPDGene African Americans using LD pruning with an r^2 threshold of 0.1 and window size of 0.5 Mb.
Each panel plots the absolute value (abs) of the correlation between principal components and genotypes on the y-axis versus the position along the genome on the x-axis. Panels are organized vertically according to which PC is being investigated (1, 2, 3, 4).

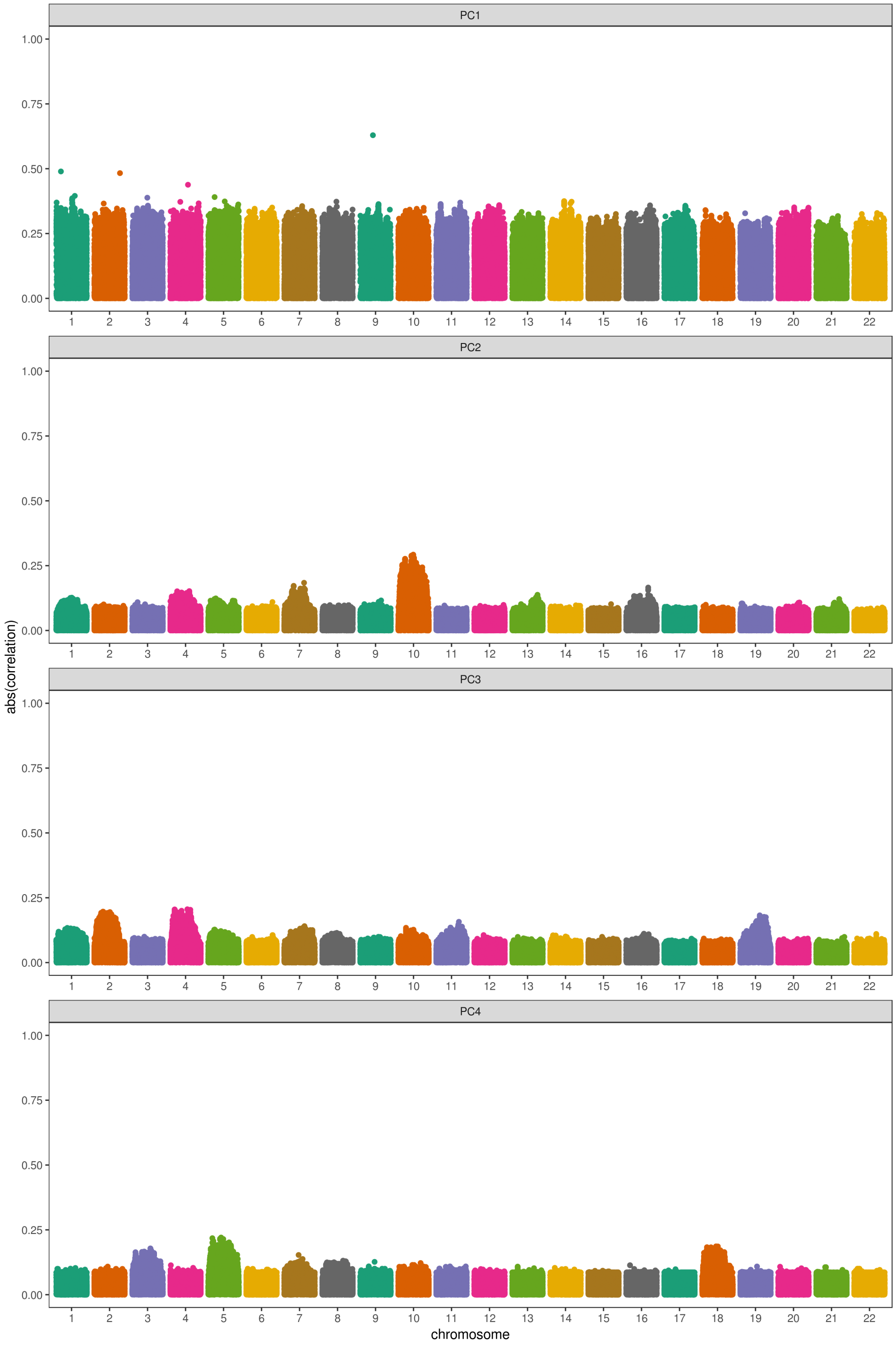

Fig B.7. Correlation between PCs and genotypes in COPDGene African Americans using LD pruning with an r^2 threshold of 0.1 and window size of 10 Mb.
Each panel plots the absolute value (abs) of the correlation between principal components and genotypes on the y-axis versus the position along the genome on the x-axis. Panels are organized vertically according to which PC is being investigated (1, 2, 3, 4).

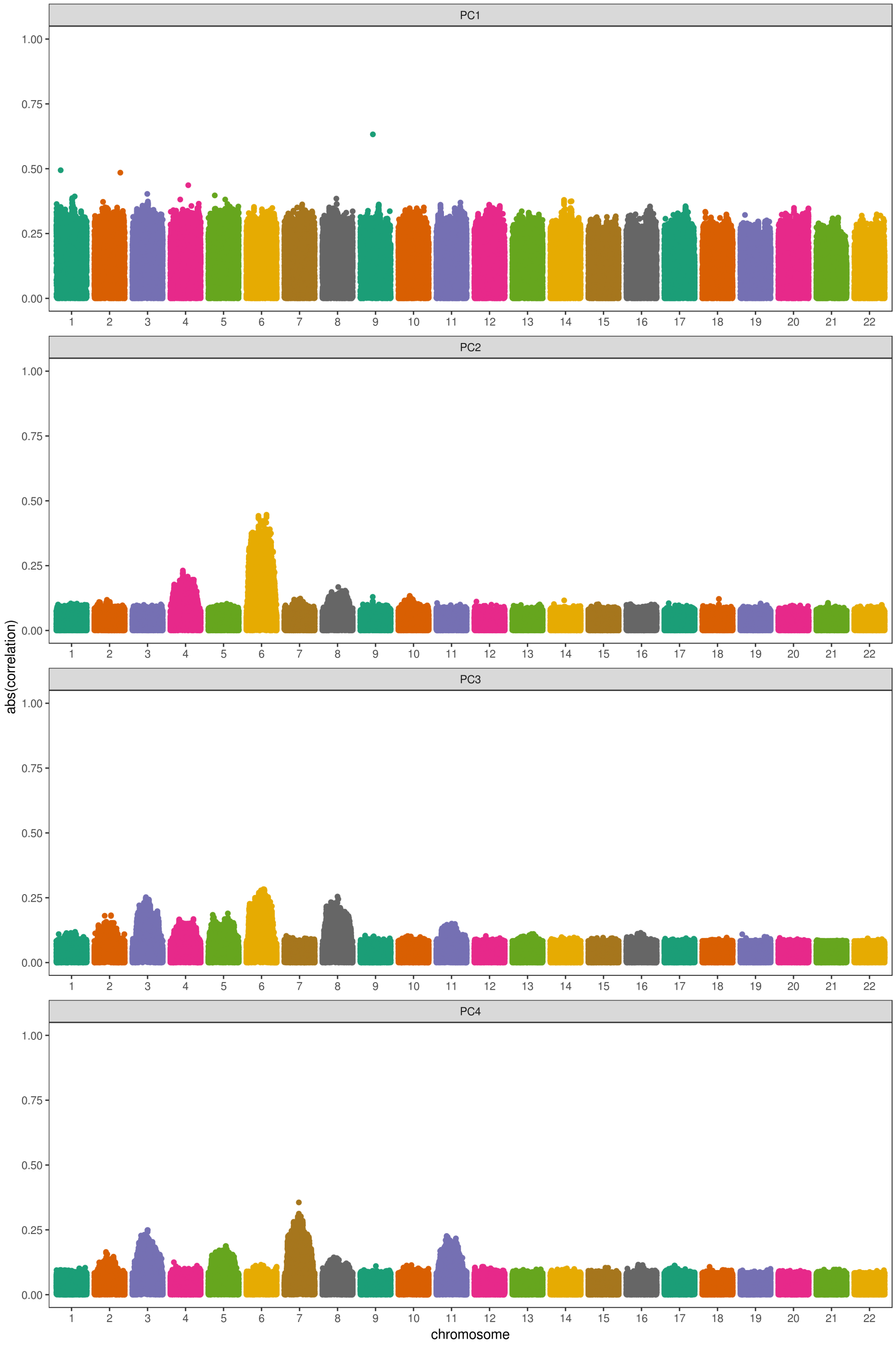

Fig B.8. Correlation between PCs and genotypes in COPDGene African Americans using LD pruning with an r^2 threshold of 0.05 and window size of 0.5 Mb.

Each panel plots the absolute value (abs) of the correlation between principal components and genotypes on the y-axis versus the position along the genome on the x-axis. Panels are organized vertically according to which PC is being investigated (1, 2, 3, 4).

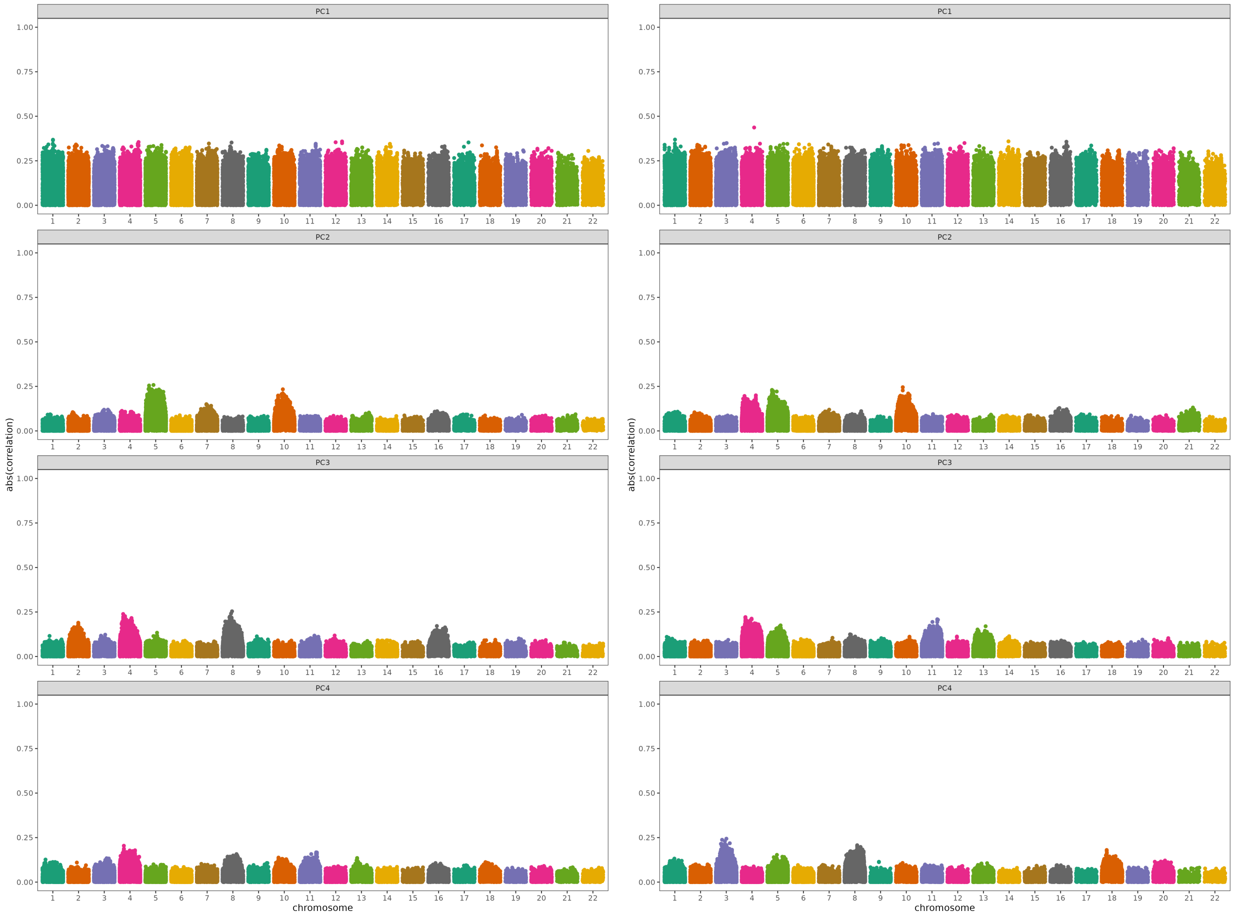

Fig B.9. Correlation between PCs and genotypes in COPDGene African Americans using LD pruning with an r^2 threshold of 0.1, window size of 10 Mb, and a stricter minor allele frequency cut-off of 2.5% (left panel) or 5% (right panel).

Each panel plots the absolute value (abs) of the correlation between principal components and genotypes on the y-axis versus the position along the genome on the x-axis. Panels are organized vertically according to which PC is being investigated (1, 2, 3, 4).

B.2.2 Proportion of Variance Explained

Across all pre-processing choices, we observe that the first PC explains a considerably higher proportion of variance than the others. The following *scree plots* present the proportion of variance explained by each PC for all sets of PCs presented in the main paper or above.

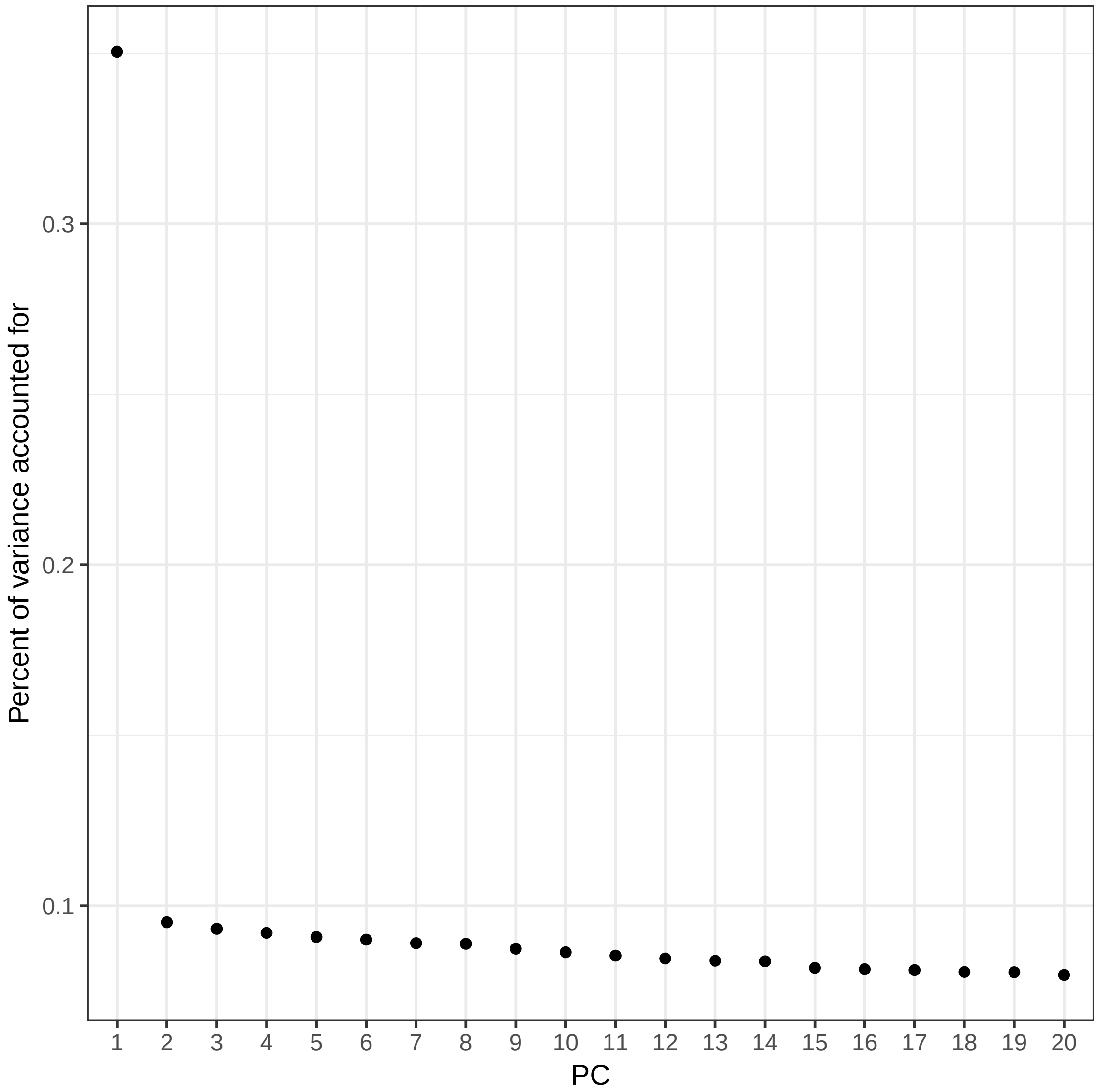

Fig B.10. Scree plot for PCs generated without any LD-based pruning or filtering in JHS.

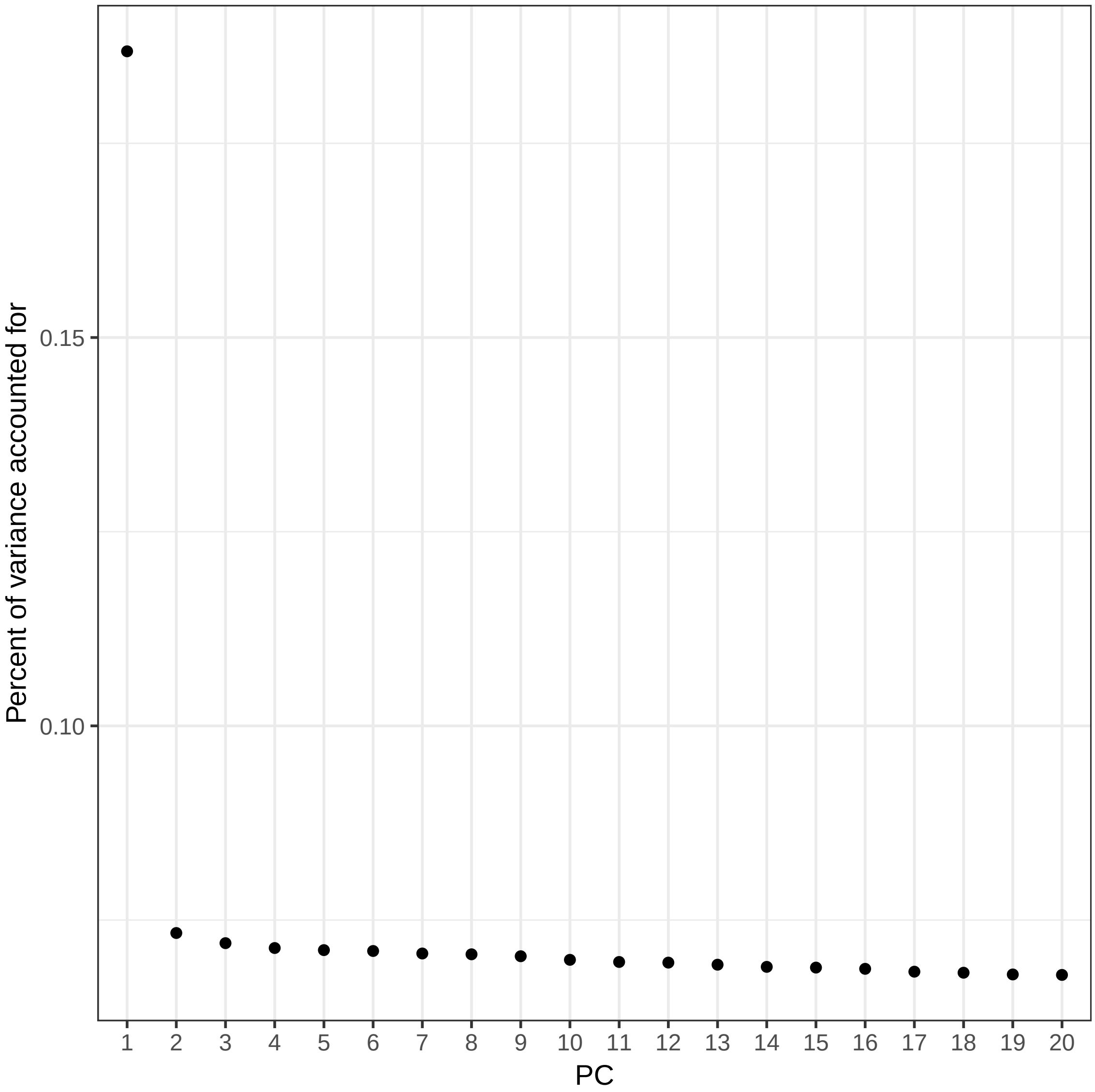

Fig B.11. Scree plot for PCs generated after LD pruning with an r^2 threshold of 0.1 and window size of 0.5 Mb in JHS.

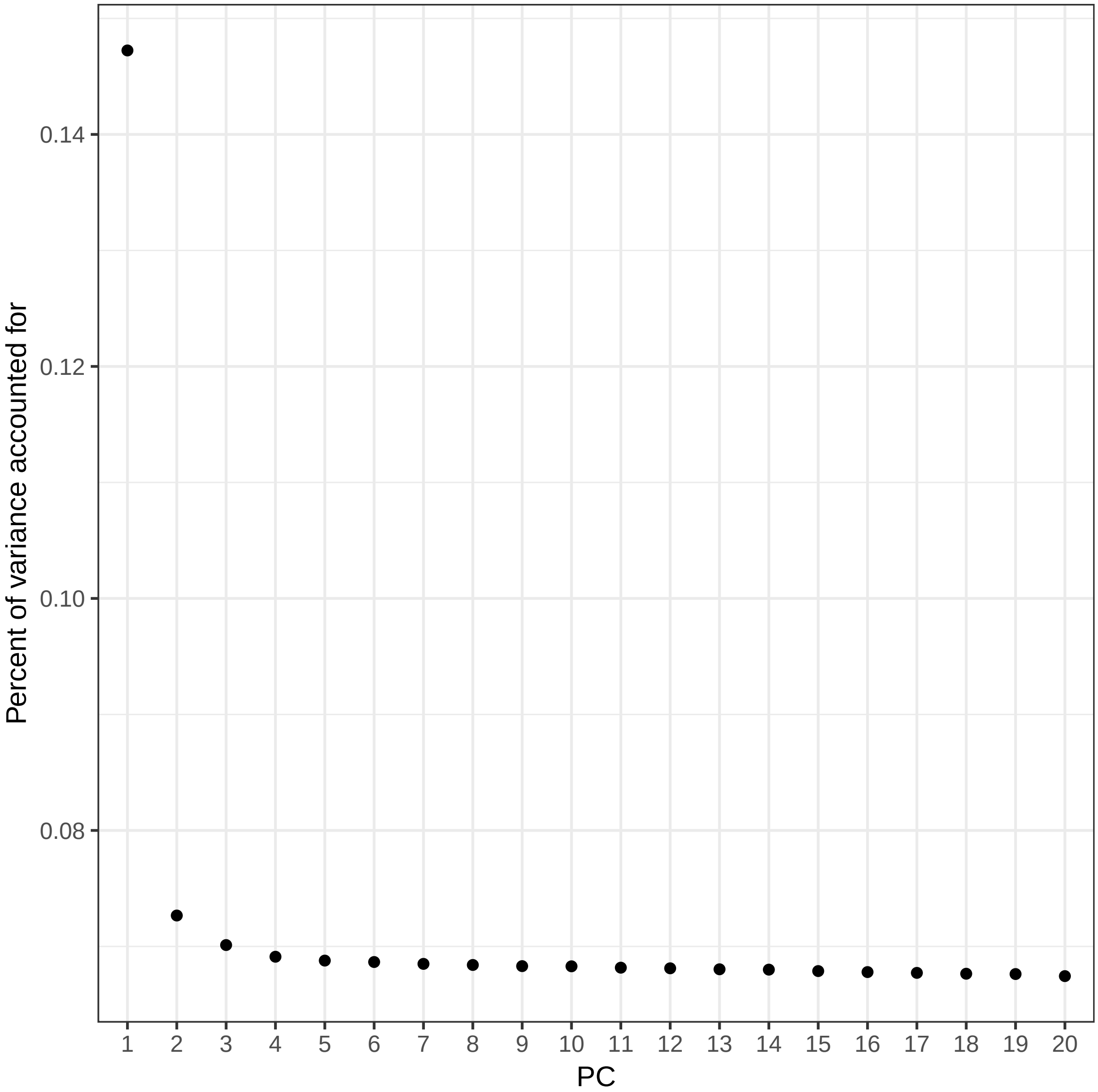

Fig B.12. Scree plot for PCs generated after LD pruning with an r^2 threshold of 0.1 and window size of 10 Mb in JHS.

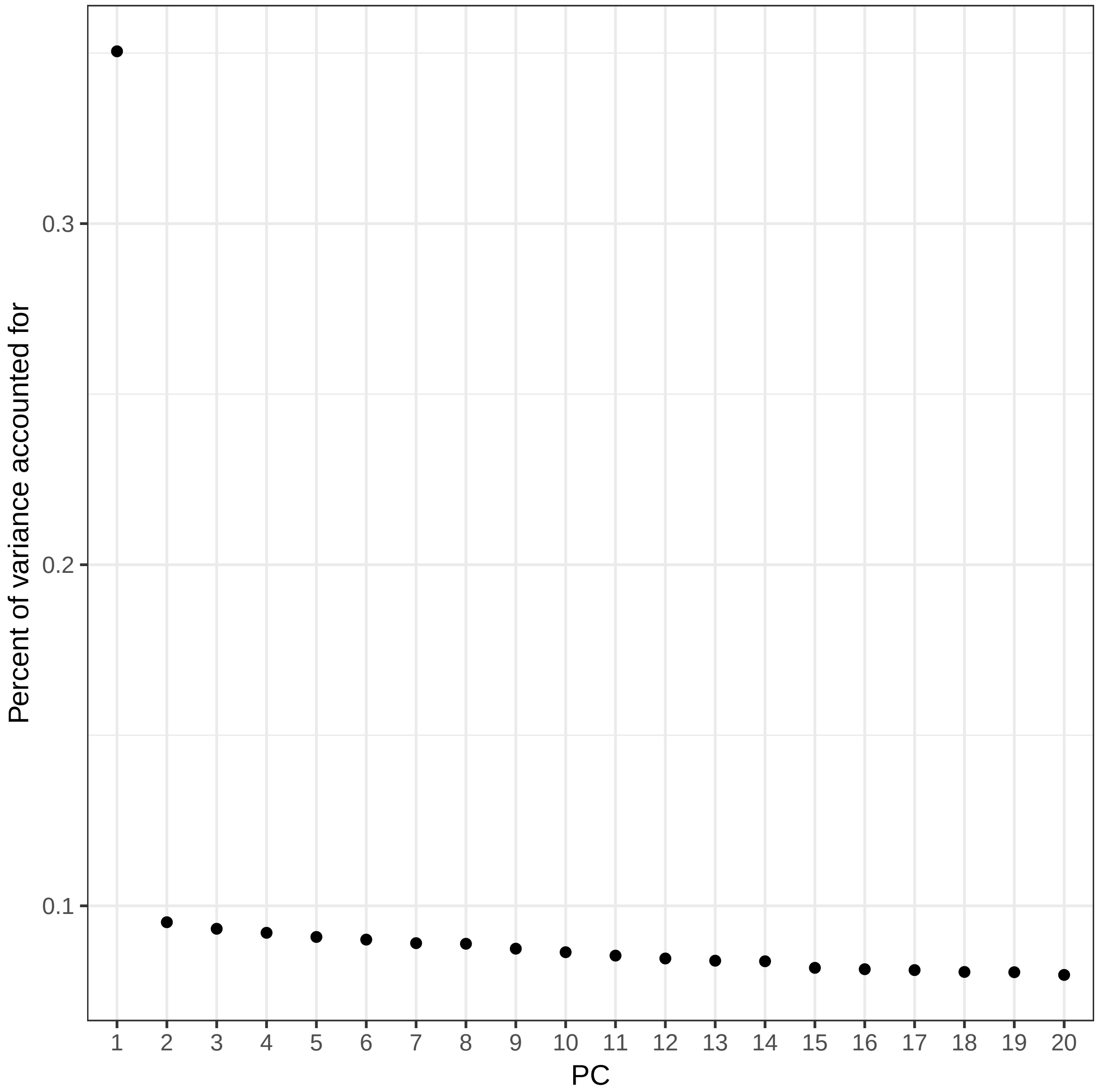

Fig B.13. Scree plot for PCs generated without any LD-based pruning or filtering in COPDGene African Americans.

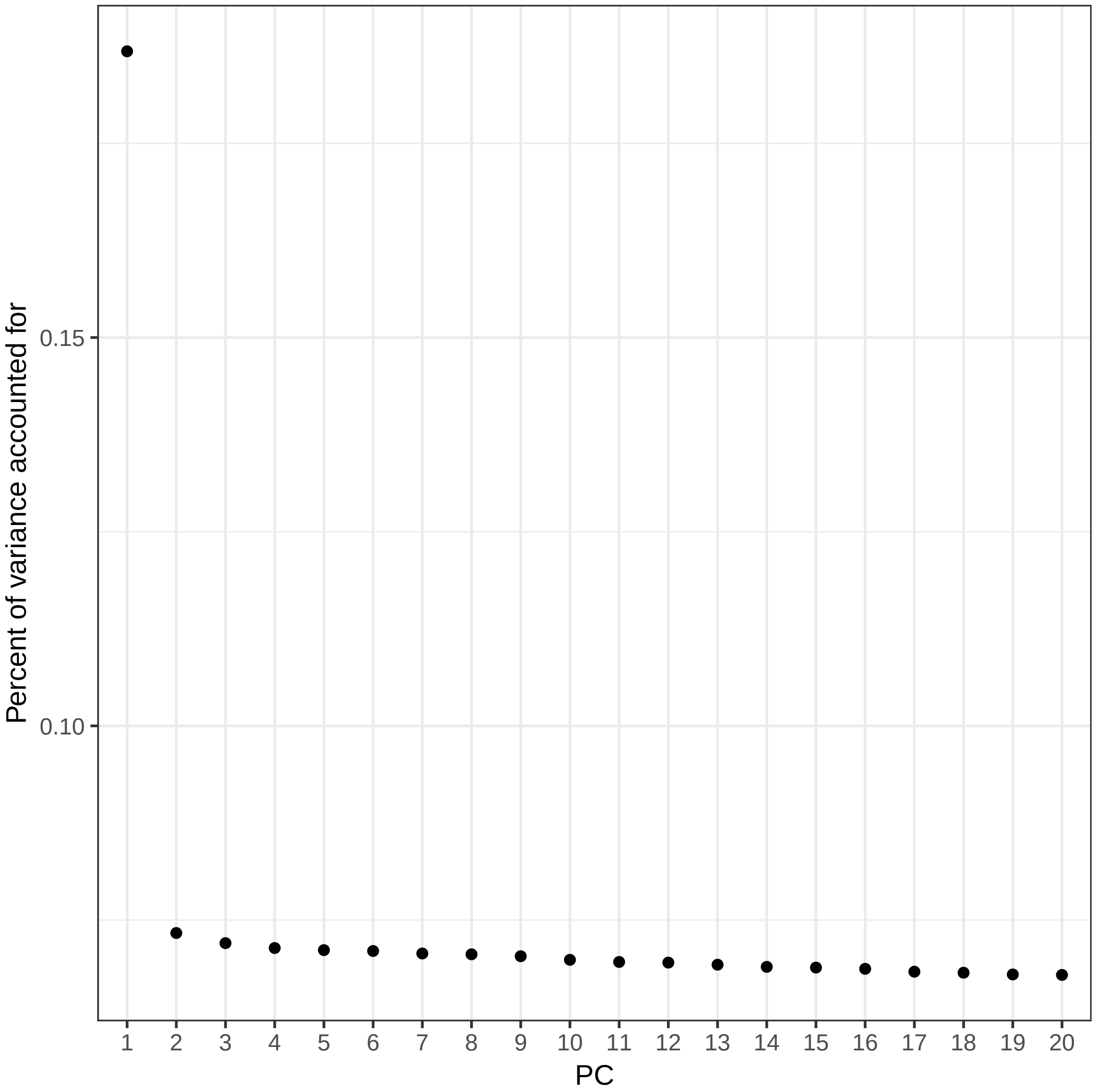

Fig B.14. Scree plot for PCs generated after LD pruning with an r^2 threshold of 0.1 and window size of 0.5 Mb in COPDGene.

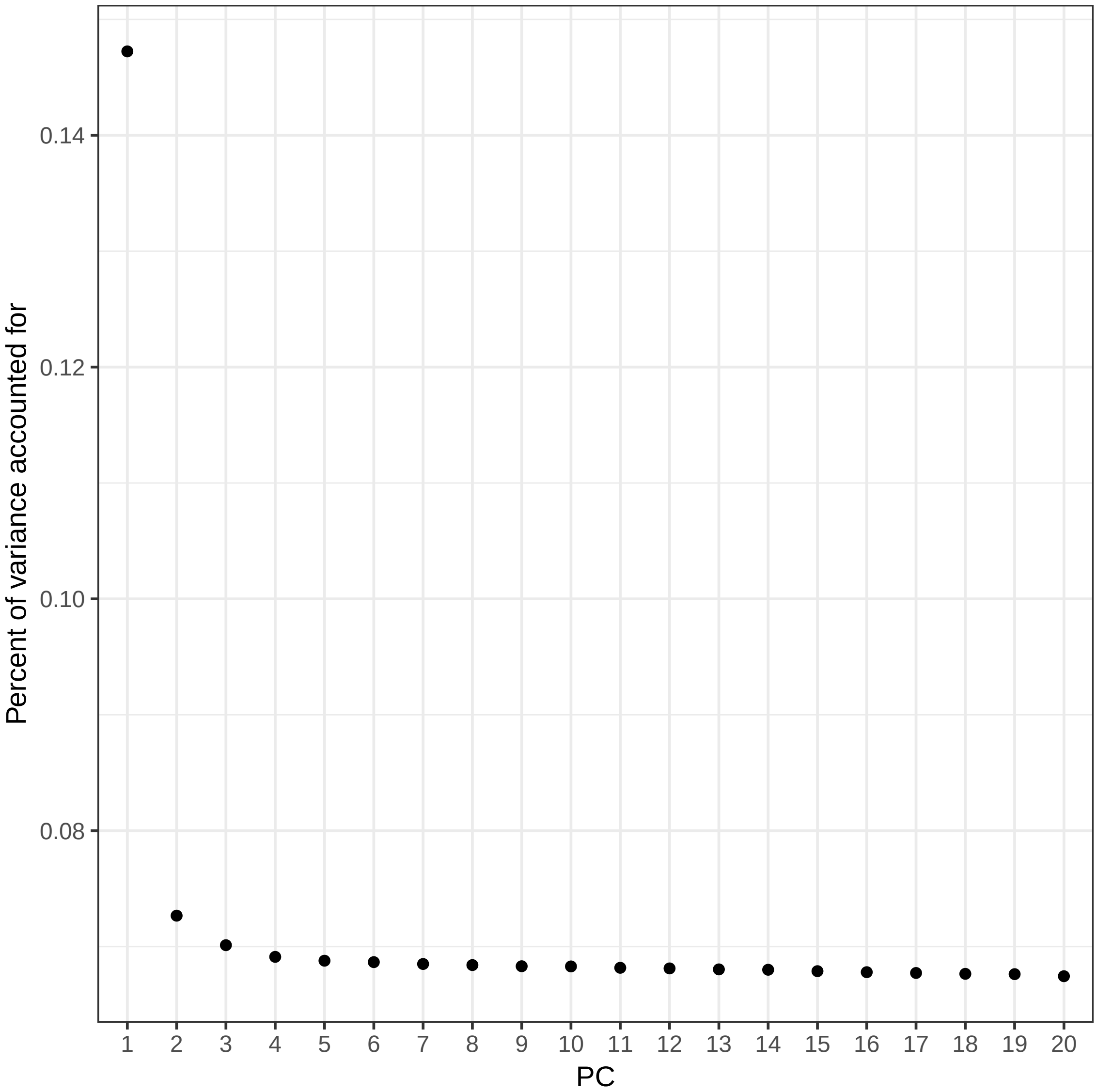

Fig B.15. Scree plot for PCs generated after LD pruning with an r^2 threshold of 0.1 and window size of 10 Mb in COPDGene.

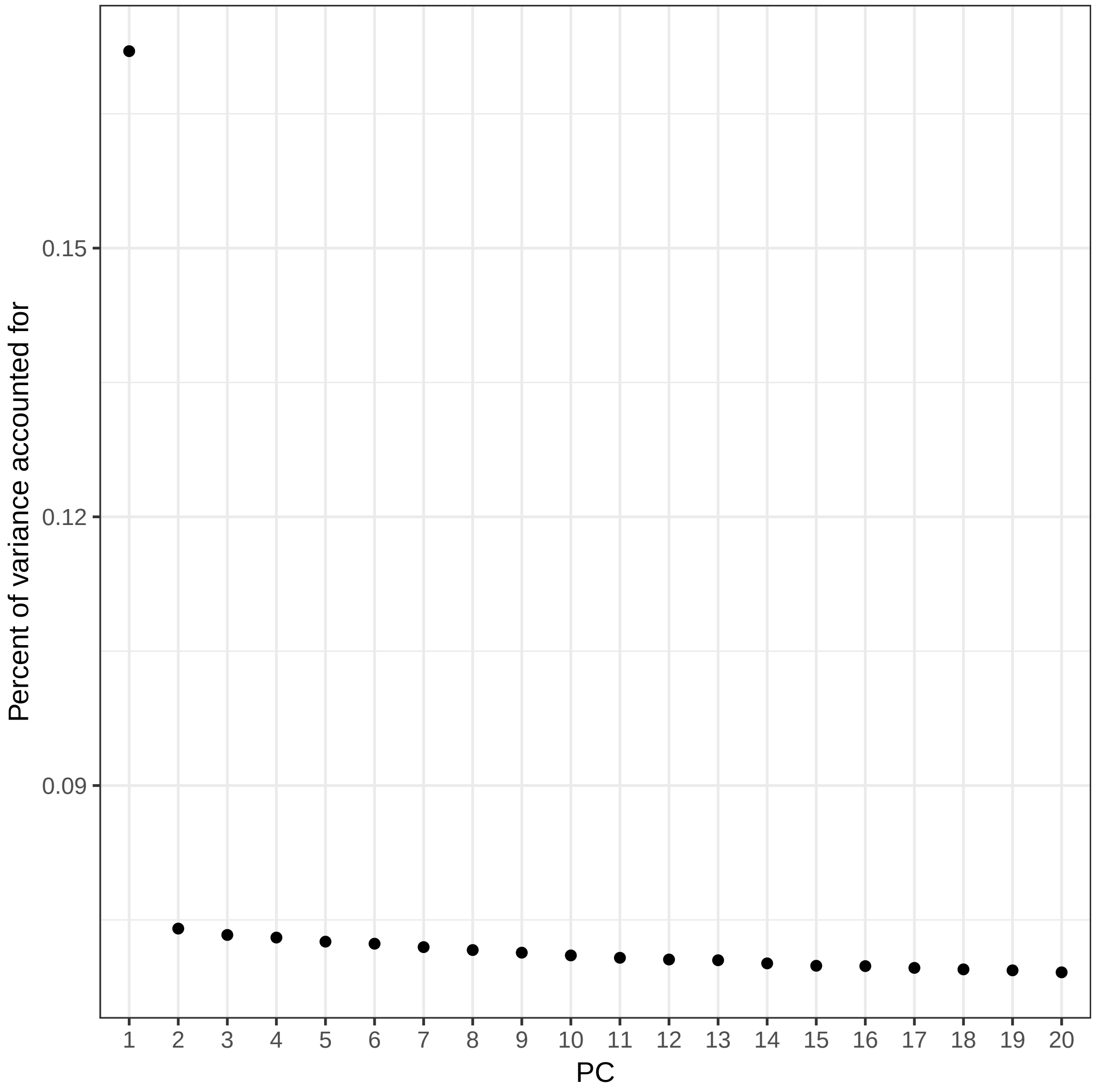

Fig B.16. Scree plot for PCs generated after LD pruning with an r^2 threshold of 0.05 and window size of 0.5 Mb in COPDGene.

C Investigation of PCs in a European American Population

We have shown that principal components can capture multiple local genomic features, rather than genome-wide ancestry, unless careful pre-processing is performed prior to running PCA. This observation is not in itself novel, but note that the patterns we observe in WHI SHARe, JHS, and COPDGene African Americans differ from what has previously been observed in European populations. In particular, in European populations a principal component might capture variation on a single chromosome[4,11] whereas in these admixed populations we see PCs driven by contributions from variants across several chromosomes. Although the focus of our work has been on admixed individuals, we were also able to run PCA on a sample of individuals with European ancestry using the COPDGene European Americans that we had excluded from our primary analyses. In this sample, we see patterns similar to those observed by previous authors, with the second and third principal components driven primarily by variants on a single chromosome: chromosome 11 (Fig C.1). This difference in what is captured by principal components in European populations versus admixed populations (i.e., variants on one chromosome versus multiple) has important implications: only when a PC captures *multiple* local genomic features does the possibility of collider bias arise. Thus, particular care must be taken when performing genome-wide association studies in admixed populations to ensure that models do not adjust for principal components that are highly correlated with variants on distinct chromosomes.

Just as the patterns of which, and how many, regions are captured by PCs differ between admixed and European populations, so too does the utility of the proposed solutions of LD pruning and removing known high LD regions. As we see in WHI SHARe and TOPMed African Americans, removing the regions listed in Table 1 without also performing LD pruning does not fully alleviate the issues with PCs capturing local genomic features (Fig C.2). We did remove a region on chromosome 11 that is listed in Table 1, but the peaks remain on

this chromosome in the SNP loadings plots for the second and third PCs. Fig C.3 presents the SNP loadings for the top four PCs after performing both these literature-based exclusions and LD pruning with default settings ($r^2 < 0.2$, window size 0.5 Mb). In contrast to what we observed above in the three admixed samples, these default LD pruning settings do seem to be sufficient in this sample of European American individuals.

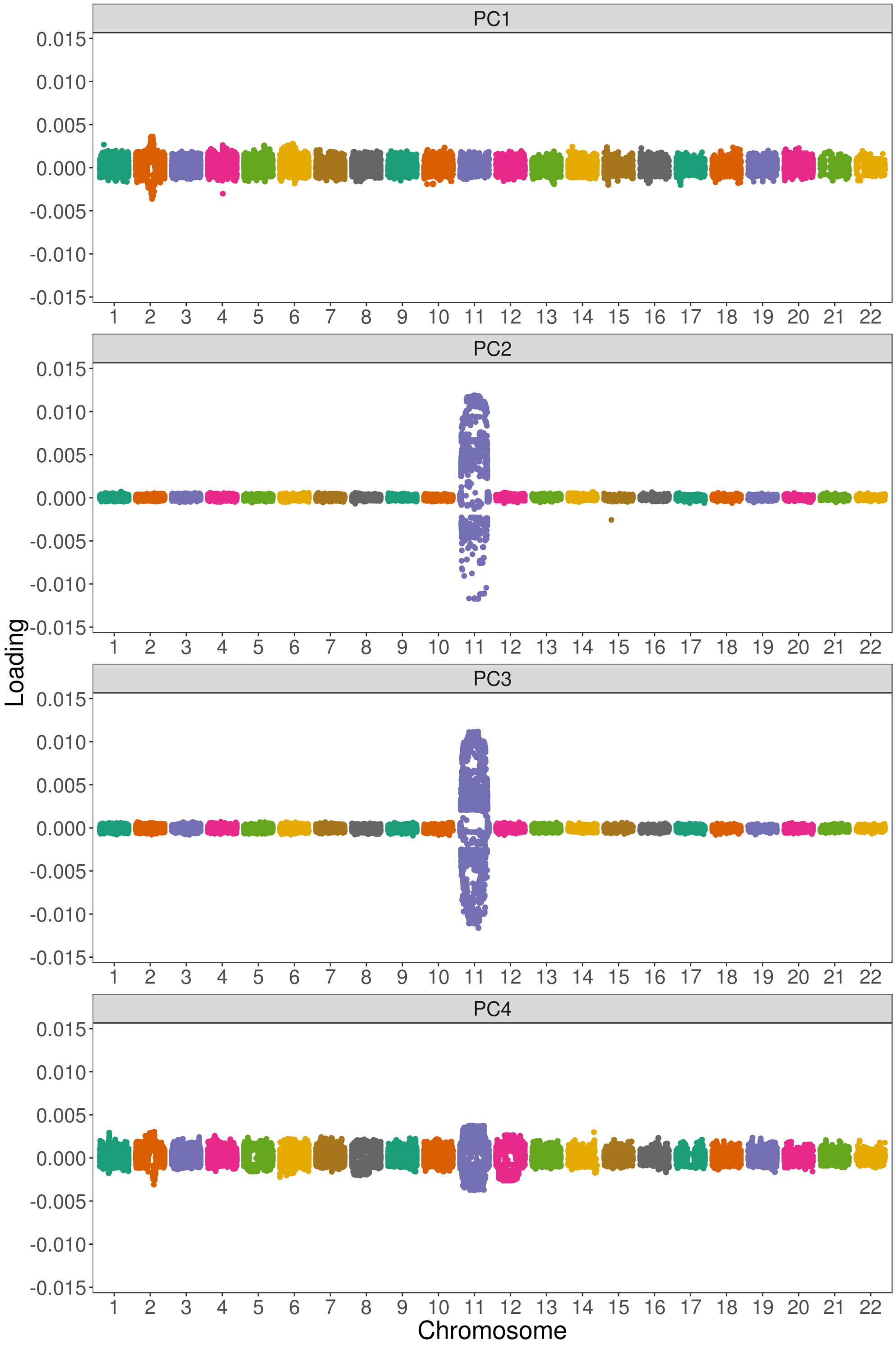

Fig C.1. SNP loadings for naively generated PCs in COPDGene European Americans. Each panel plots the principal component loading (y-axis) versus the position along the genome (x-axis) for each variant. Panels are organized vertically according to which PC is being investigated (1, 2, 3, 4). Unlike in admixed populations, we see a single peak on chromosome 11.

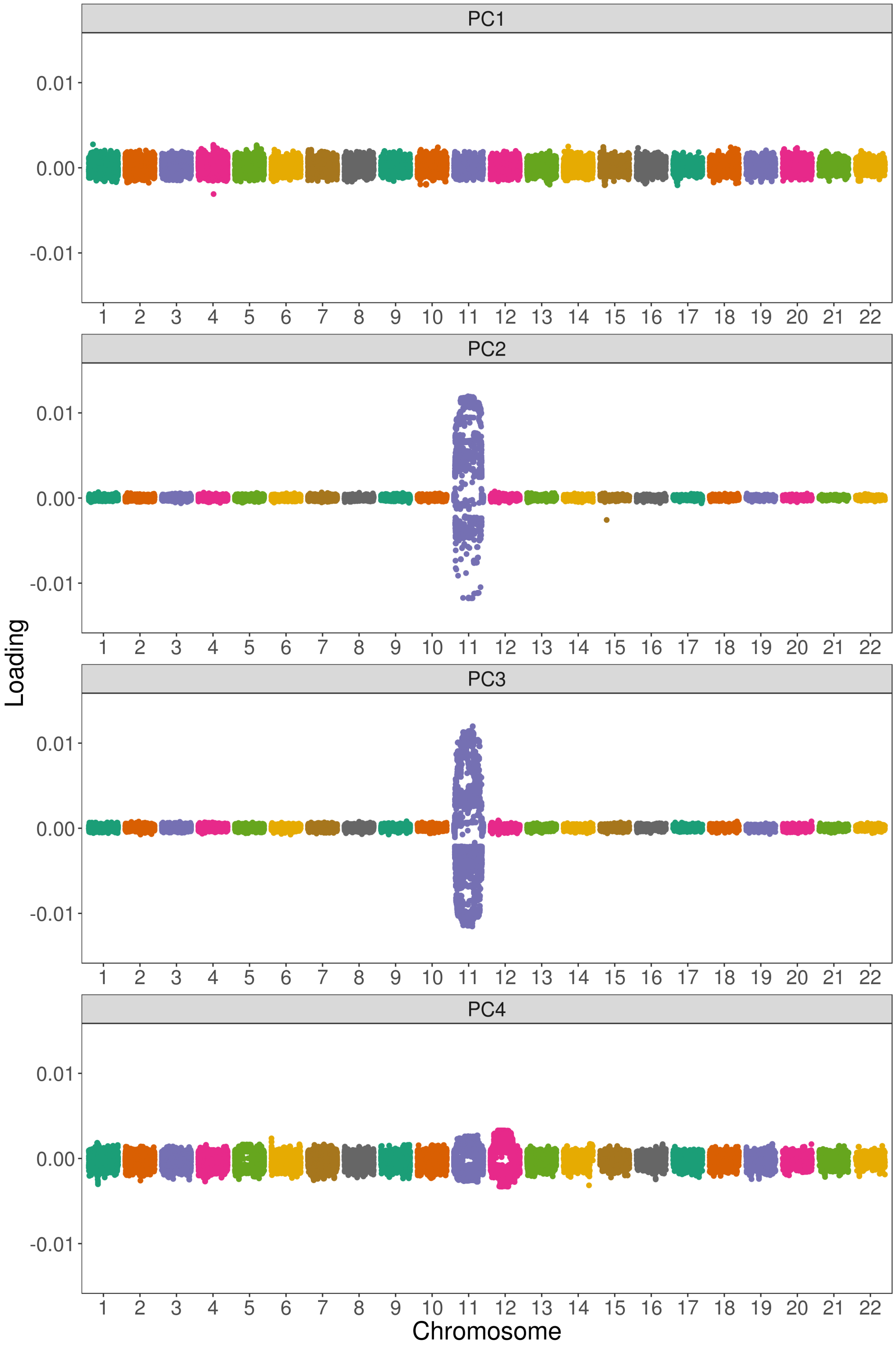

Fig C.2. SNP loadings for PCs in COPDGene European Americans after removing regions listed in Table 1, but no LD pruning.
Each panel plots the principal component loading (y-axis) versus the position along the genome (x-axis) for each variant. Panels are organized vertically according to which PC is being investigated (1, 2, 3, 4). Some smaller peaks (e.g., the higher loadings on chromsome 2 for the first and fourth PCs) are gone, but the major peaks on chromosome 11 remain.

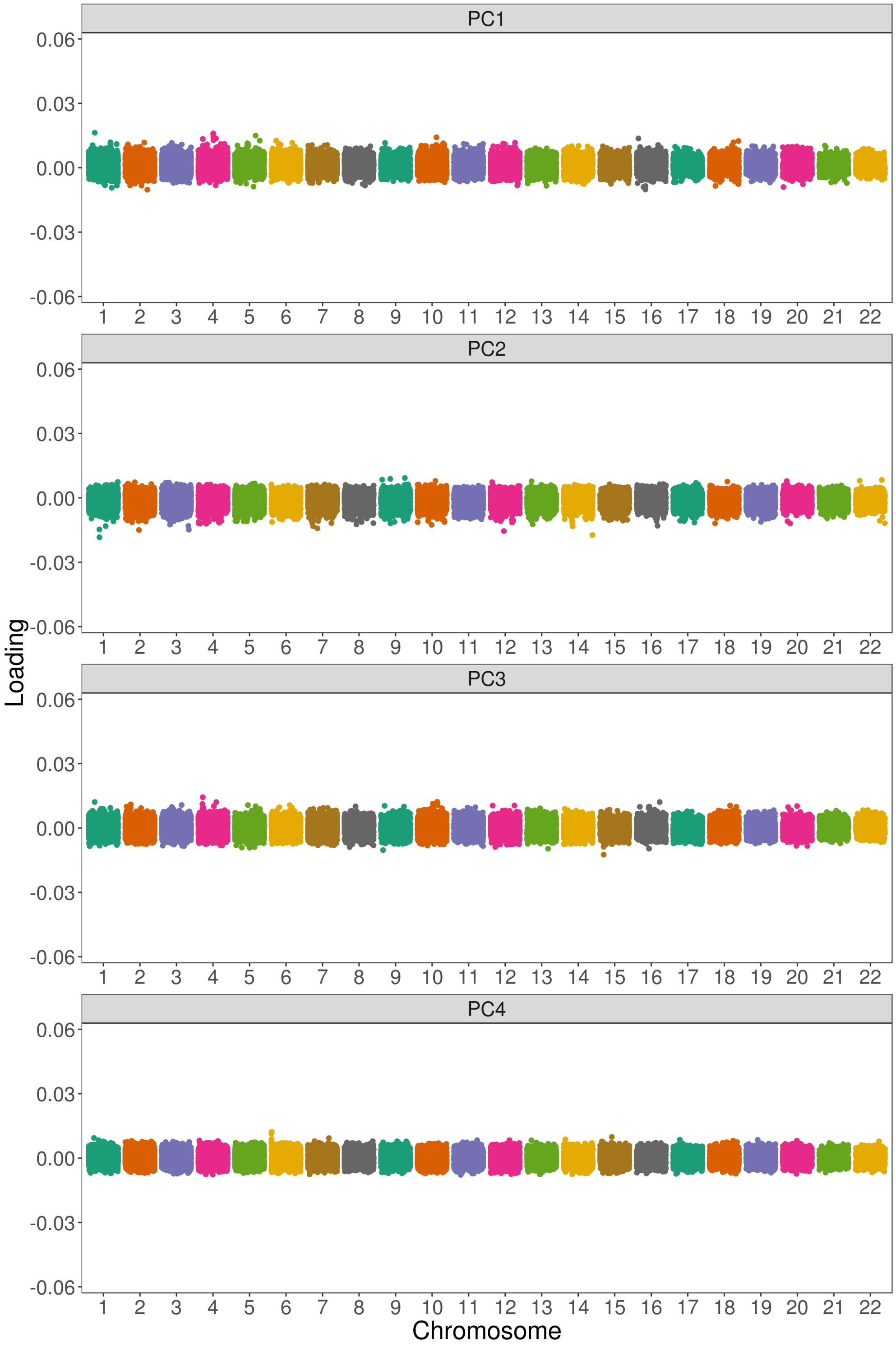

Fig C.3. SNP loadings for PCs in COPDGene European Americans after removing regions listed in Table 1 and performing LD pruning with typical default settings ($r^2 < 0.2$, window size = 0.5 Mb).

Each panel plots the principal component loading (y-axis) versus the position along the genome (x-axis) for each variant. Panels are organized vertically according to which PC is being investigated (1, 2, 3, 4). Unlike in admixed populations, here the default LD pruning settings are sufficient in preventing PCs from capturing local genomic features.

D Comparison of PCs and Model-Based Admixture Proportions

In many African American populations, only one principal component may be needed to capture ancestral heterogeneity, at least with respect to differences in the relative proportion of African and European continental ancestry. We investigated whether this statement holds true in three samples of African American individuals from the Women's Health Initiative SNP Health Association Resource (WHI SHARe) and two Trans-Omics for Precision Medicine (TOPMed) contributing studies: the Jackson Heart Study (JHS) and the Chronic Obstructive Pulmonary Disease Genetic Epidemiology Study (COPDGene). Comparing model-based admixture proportions (estimated using `RFMix`[12] in WHI SHARe and an unsupervised `ADMIXTURE`[13] analysis in JHS and COPDGene) to principal components shows that the first PC is in fact highly correlated with the inferred proportion of African ancestry in these samples, while later PCs show very little correlation with genome-wide continental ancestry. This pattern holds regardless of whether PCs are generated with (Fig D.2) or without (Fig D.1) prior filtering and pruning based on linkage disequilibrium (LD).

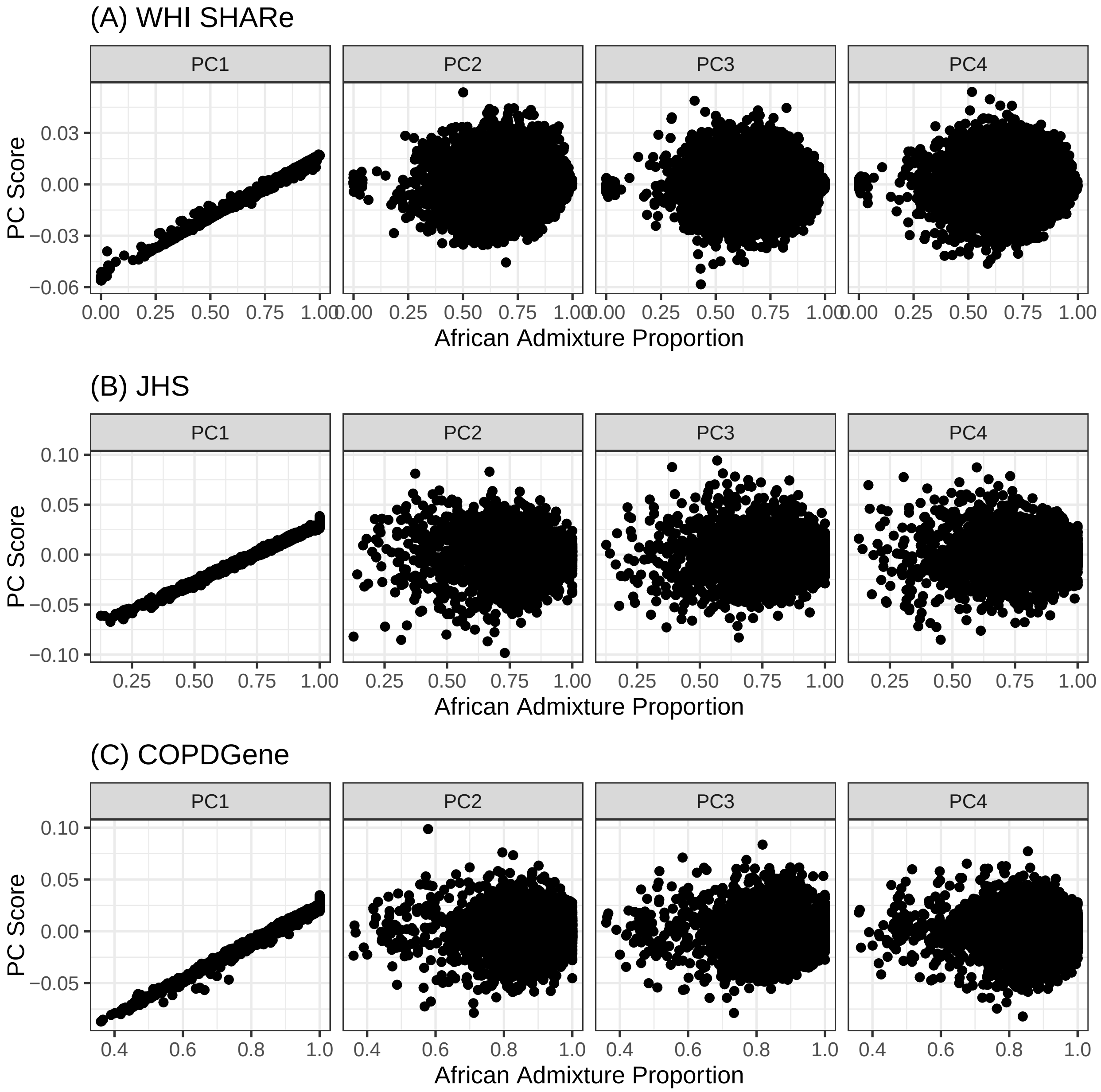

Fig D.1. Scatterplots of estimated African admixture proportions versus the first four PCs in (A) WHI SHARe, (B) TOPMed JHS, and (C) TOPMed COPDGene African Americans. Here we consider PCs that were generated on the entire set of SNPs (i.e., without any prior LD-based filtering or pruning).

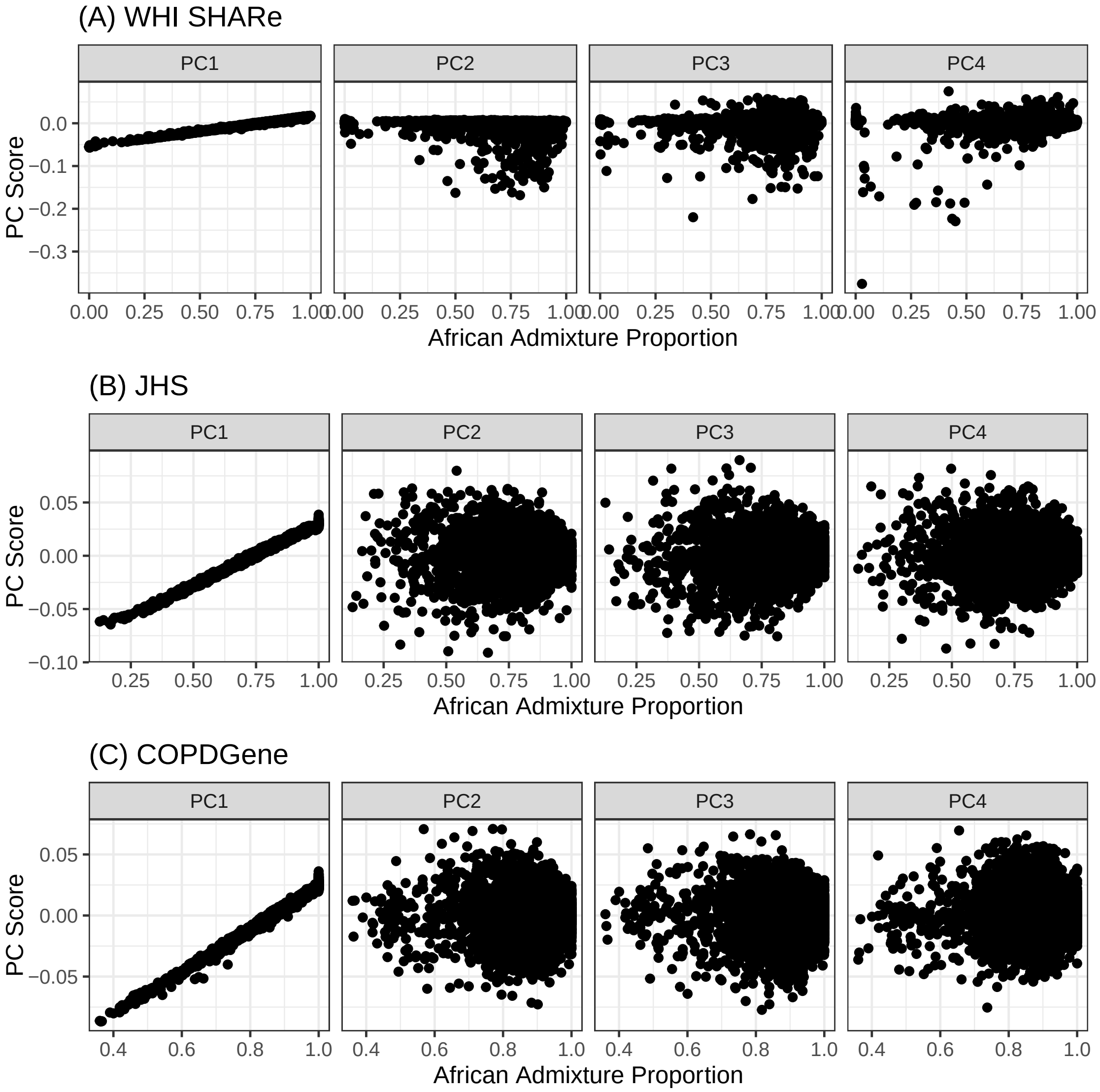

Fig D.2. Scatterplots of estimated African admixture proportions versus the first four PCs in (A) WHI SHARe, (B) TOPMed JHS, and (C) TOPMed COPDGene African Americans. Here we consider PCs that were generated after both LD pruning ($r^2 = 0.1$, window size = 0.5 Mb) and filtering previously identified high-LD regions (Table 1).

E GWAS Effect Size Derivations

To illustrate the impact of adjusting for extraneous PCs, particularly if those PCs capture multiple local genomic features, we derived the expected effect size estimates for GWAS models adjusting for different sets of covariates. Details, and a simulation study validating these theoretical results, are presented below.

E.1 Assumed data-generating mechanism

We consider an admixed population with two ancestral populations, n individuals, and admixture proportions $\boldsymbol{\pi}_i = \begin{pmatrix} \pi_i & 1-\pi_i \end{pmatrix}^\top$ that are allowed to vary across the population. We refer to the two ancestral populations as *Ancestral Population 1* and *Ancestral Population 2*, with π_i representing the genome-wide proportion of genetic material inherited by individual i from Ancestral Population 1 and $1-\pi_i$ representing the proportion of genetic material inherited from Ancestral Population 2. We denote local ancestry by $\mathbf{a}_{ij} = \begin{pmatrix} a_{ij} & 2-a_{ij} \end{pmatrix}^\top$, where a_{ij} and $2-a_{ij}$ are the number of alleles inherited by individual i from Ancestral Populations 1 and 2, respectively, at position j. Genotypes, quantified as the number of copies of some pre-specified allele carried by individual i at position j, are represented by g_{ij}. We consider two *unlinked* variants $j = 1, 2$ (e.g., variants on distinct chromosomes) and assume that data are generated according to the following hierarchical model:

$$\begin{aligned}
\pi_i &\overset{\text{i.i.d.}}{\sim} F \text{ for some distribution } F \\
a_{ij} \mid \pi_i &\overset{\text{i.i.d}}{\sim} \text{Binomial}(2, \pi_i), \;\; j = 1, 2 \\
g_{ij} \mid a_{ij}, \mathbf{p}_j &\overset{\text{ind.}}{\sim} \text{Binomial}(a_{ij}, p_{j1}) + \text{Binomial}(2 - a_{ij}, p_{j2}), \;\; j = 1, 2
\end{aligned}$$

where p_{j1}, p_{j2} are allele frequencies at position j in Ancestral Populations 1 and 2, respectively. Since the two variants under consideration are unlinked, we assume that local ancestry and genotypes at these positions are conditionally independent.

We assume that our quantitative trait of interest $\mathbf{y}$ depends only on the genotype at position 1 ($j = 1$), and we allow for the possibility that the admixture proportions $\boldsymbol{\pi}$ have a direct effect on the trait (e.g., through environmental differences across ancestral populations). More specifically, we assume that this trait is generated according to

$$y_i = \beta_0 + \beta_1 g_{i1} + \beta_\pi \pi_i + \epsilon_i, \quad \epsilon_i \overset{\text{i.i.d}}{\sim} (0, \sigma_\epsilon^2).$$

We refer to β_1 and β_2 as the true *effect sizes* of variants 1 and 2, respectively. Since the trait only depends on the genotype at position 1, the true effect size of position 2 is $\beta_2 = 0$.

Assuming that data are generated according to the above-described mechanisms, and defining $E_\pi := E(\pi)$ and $V_\pi := \text{Var}(\pi)$, then the following statements are true. For notational simplicity, we drop the subscript i.

- $E(a_j) = 2E_\pi, \ j = 1, 2$
- $V(a_j) = 2\{V_\pi + E_\pi(1 - E_\pi)\}, \ j = 1, 2$
- $\text{Cov}(a_1, a_2) = 4V_\pi$
- $\text{Cov}(a_j, \pi) = 2V_\pi, \ j = 1, 2$
- $E(g_j) = 2\{p_{j2} + (p_{j1} - p_{j2})E_\pi\}, \ j = 1, 2$
- $V(g_j) = 2[p_{j2}(1-p_{j2})+(p_{j1}-p_{j2})(1-p_{j1}-p_{j2})E_\pi+(p_{j1}-p_{j2})^2\{V_\pi+E_\pi(1-E_\pi)\}], \ j = 1, 2$
- $\text{Cov}(g_1, g_2) = 4(p_{11} - p_{12})(p_{21} - p_{22})V_\pi$
- $\text{Cov}(g_j, g_j) = 2(p_{j1} - p_{j2})\{V_\pi + E_\pi(1 - E\pi)\}, \ j = 1, 2$
- $\text{Cov}(g_j, g_k) = 4(p_{j1} - p_{j2})V_\pi, \ j \neq k$
- $\text{Cov}(g_j, \pi) = 2(p_{j1} - p_{j2})V_\pi, \ j = 1, 2$

Furthermore, suppose we define a random variable $z_g = h(g_1, g_2) + e, \ e \sim (\mu_e, \sigma_e^2)$ for some function h. Then:

- $E(z_g) = \mu_e + E\{h(g_1, g_2)\}$
- $V(z_g) = \sigma_e^2 + V\{h(g_1, g_2)\}$
- $\text{Cov}(\pi, z_g) = \text{Cov}[\pi, E\{h(g_1, g_2) \mid \pi\}]$
- $\text{Cov}(a_j, z_g) = 2\text{Cov}(\pi, z_g) + E[\text{Cov}\{a_j, h(g_1, g_2) \mid \pi\}],\ j = 1, 2$
- $\text{Cov}(g_j, z_g) = 2(p_{j1} - p_{j2})\text{Cov}(\pi, z_x) + E[\text{Cov}\{g_j, h(g_1, g_2) \mid \pi\}],\ j = 1, 2$

These results are straightforward to derive, using our assumed hierarchical data-generating model and the laws of total expectation

$$E[x] = E\{E[x \mid y]\},$$

total variance

$$V[x] = V\{E[x \mid y]\} + E\{V[x \mid y]\},$$

and total covariance

$$\text{Cov}[x, y] = \text{Cov}\{E[x \mid z], E[y \mid z]\} + E\{\text{Cov}[x, y \mid z]\}.$$

E.2 Expected effect size estimates

Suppose we conduct a genome-wide association study using either an *unadjusted*, *admixture proportion adjusted*, or *principal component adjusted* GWAS model. These models can be written as follows:

$$\begin{aligned}
\text{Unadjusted: } & E[y_i \mid g_{ij}] = \alpha + \beta_j g_{ij}, \\
\text{Admixture Proportion Adjusted: } & E[y_i \mid g_{ij}, \pi_i] = \alpha + \beta_j g_{ij} + \gamma \pi_i, \\
\text{Principal Component Adjusted: } & E[y_i \mid g_{ij}, u_{1i}, \ldots, u_{pi}] = \alpha + \beta_j g_{ij} + \gamma_1 u_{1i} + \cdots + \gamma_p u_{pi},
\end{aligned}$$

for some number of PCs p. The expected effect size estimates from these models can be derived using the theory of linear models.

E.2.1 Unadjusted model

If we fit an unadjusted GWAS model, as defined above, then the estimate of the effect size of the variant at position j takes the following form in expectation:

$$E[\hat{\beta}_j] = \frac{\beta_1 \widehat{\text{Cov}}(g_1, g_j) + \beta_\pi \widehat{\text{Cov}}(\pi, g_j)}{\widehat{\text{Var}}(g_j)},$$

where $\widehat{\text{Var}}$ and $\widehat{\text{Cov}}$ are the sample variance and covariance, respectively, across all n individuals in the sample (e.g., $\widehat{\text{Var}}(g_j) = \frac{1}{n-1}\sum_{i=1}^{n}(g_{ij} - \bar{g}_j)^2$).

Proof. Let $\boldsymbol{\pi}, \mathbf{a}_1, \mathbf{a}_2, \mathbf{g}_1, \mathbf{g}_2$ be drawn from the hierarchical model specified above. Assume that the trait $\mathbf{y}$ is generated such that $\mathbf{y} = \beta_0 \mathbf{1} + \beta_1 \mathbf{g}_1 + \beta_\pi \boldsymbol{\pi} + \boldsymbol{\epsilon}$, where ϵ_i are drawn *i.i.d.* from some distribution with mean 0 and variance σ_ϵ^2. Suppose that at position j we fit the unadjusted GWAS model $E[\mathbf{y} \mid \mathbf{g}_j] = \beta_0 \mathbf{1} + \beta_j \mathbf{g}_j$. Then, the estimated regression coefficients for this model will take the form

$$\hat{\boldsymbol{\beta}}_j = \begin{pmatrix} \hat{\beta}_0 \\ \hat{\beta}_j \end{pmatrix} = (\mathbf{X}^\top \mathbf{X})^{-1} \mathbf{X}^\top \mathbf{y}, \text{ for } \mathbf{X} = \begin{pmatrix} \mathbf{1} & \mathbf{g}_j \end{pmatrix},$$

with expected value

$$E[\hat{\boldsymbol{\beta}}_j] = (\mathbf{X}^\top \mathbf{X})^{-1} \mathbf{X}^\top \mathbf{X}^* \boldsymbol{\beta}, \text{ for } \mathbf{X}^* = \begin{pmatrix} \mathbf{1} & \mathbf{g}_1 & \boldsymbol{\pi} \end{pmatrix} \text{ and } \boldsymbol{\beta} = \begin{pmatrix} \beta_0 \\ \beta_1 \\ \beta_\pi \end{pmatrix}.$$

But

$$(\mathbf{X}^\top \mathbf{X})^{-1} = \begin{pmatrix} \mathbf{1}^\top \mathbf{1} & \mathbf{1}^\top \mathbf{g}_j \\ \mathbf{g}_j^\top \mathbf{1} & \mathbf{g}_j^\top \mathbf{g}_j \end{pmatrix}^{-1} = \frac{1}{n\widehat{\text{Var}}(g_j)} \begin{pmatrix} \widehat{\text{Var}}(g_j) + \hat{E}(g_j)^2 & -\hat{E}(g_j) \\ -\hat{E}(g_j) & 1 \end{pmatrix}$$

and

$$\mathbf{X}^\top \mathbf{X}^* = \begin{pmatrix} \mathbf{1}^\top \mathbf{1} & \mathbf{1}^\top \mathbf{g}_1 & \mathbf{1}^\top \boldsymbol{\pi} \\ \mathbf{g}_j^\top \mathbf{1} & \mathbf{g}_j^\top \mathbf{g}_1 & \mathbf{g}_j^\top \boldsymbol{\pi} \end{pmatrix} = n \begin{pmatrix} 1 & \hat{E}(g_1) & \hat{E}(\pi) \\ \hat{E}(g_j) & \widehat{\mathrm{Cov}}(g_1, g_j) + \hat{E}(g_1)\hat{E}(g_j) & \widehat{\mathrm{Cov}}(\pi, g_j) + \hat{E}(\pi)\hat{E}(g_j) \end{pmatrix}.$$

It follows that

$$E[\hat{\boldsymbol{\beta}}_j] = \frac{1}{\widehat{\mathrm{Var}}(g_j)} \begin{pmatrix} \widehat{\mathrm{Var}}(g_j) & \widehat{\mathrm{Var}}(g_j)\hat{E}(g_1) - \widehat{\mathrm{Cov}}(g_j, g_1)\hat{E}(g_j) & \widehat{\mathrm{Var}}(g_j)\hat{E}(\pi) - \hat{E}(g_j)\widehat{\mathrm{Cov}}(g_j, \pi) \\ 0 & \widehat{\mathrm{Cov}}(g_j, g_1) & \widehat{\mathrm{Cov}}(g_j, \pi) \end{pmatrix} \boldsymbol{\beta},$$

and thus

$$E[\hat{\beta}_j] = \frac{\beta_1 \widehat{\mathrm{Cov}}(g_j, g_1) + \beta_\pi \widehat{\mathrm{Cov}}(\pi, g_j)}{\widehat{\mathrm{Var}}(g_j)},$$

as desired. □

Using the results from Section E.1, we can simplify this result further. We will consider studies with large sample sizes, such that we can replace the sample variance $\widehat{\mathrm{Var}}$ and covariance $\widehat{\mathrm{Cov}}$ with their population equivalent. At the causal variant (position $j = 1$), the expected effect size estimate becomes

$$\begin{aligned} E[\hat{\beta}_1] &= \frac{\beta_1 \mathrm{Cov}(g_1, g_1) + \beta_\pi \mathrm{Cov}(\pi, g_1)}{\mathrm{Var}(g_1)} \\ &= \frac{\beta_1 \mathrm{Var}(g_1) + \beta_\pi \mathrm{Cov}(\pi, g_1)}{\mathrm{Var}(g_1)} \\ &= \beta_1 + \frac{\beta_\pi V_\pi (p_{11} - p_{12})}{p_{12}(1 - p_{12}) + (p_{11} - p_{12})(1 - p_{11} - p_{12})E_\pi + (p_{11} - p_{12})^2 (V_\pi + E_\pi - E_\pi^2)}, \end{aligned} \tag{E.1}$$

and at the unlinked neutral variant (position $j = 2$),

$$\begin{aligned} E[\hat{\beta}_2] &= \frac{\beta_1 \mathrm{Cov}(g_1, g_2) + \beta_\pi \mathrm{Cov}(\pi, g_2)}{\mathrm{Var}(g_2)} \\ &= \frac{(p_{21} - p_{22}) V_\pi [2\beta_1 (p_{11} - p_{12}) + \beta_\pi]}{p_{22}(1 - p_{22}) + (p_{21} - p_{22})(1 - p_{21} - p_{22})E_\pi + (p_{21} - p_{22})^2 (V_\pi + E_\pi - E_\pi^2)}. \end{aligned} \tag{E.2}$$

E.2.2 Admixture proportion adjusted model

Now suppose that we fit a GWAS model that adjusts for the true admixture proportions, π_i. The expected effect size estimate at variant j is:

$$E[\hat{\beta}_j] = \beta_1 \frac{\widehat{\text{Var}}(\pi)\widehat{\text{Cov}}(g_1, g_j) - \widehat{\text{Cov}}(g_1, \pi)\widehat{\text{Cov}}(g_j, \pi)}{\widehat{\text{Var}}(\pi)\widehat{\text{Var}}(g_j) - \widehat{\text{Cov}}(g_j, \pi)^2}$$

Proof. The proof of this result follows similar arguments to that for the unadjusted model, replacing the design matrix $\mathbf{X}$ with $\begin{pmatrix} \mathbf{1} & \mathbf{g}_j & \boldsymbol{\pi} \end{pmatrix}$. With a bit of algebra, the rest follows. □

Again, we can simplify this result further using the results from Section E.1 and the assumption of large sample sizes. At the causal variant (position $j = 1$), the expected effect size estimate simplifies to

$$\begin{aligned} E[\hat{\beta}_1] &= \beta_1 \frac{\text{Var}(\pi)\text{Cov}(g_1, g_1) - \text{Cov}(g_1, \pi)\text{Cov}(g_1, \pi)}{\text{Var}(\pi)\text{Var}(g_1) - \text{Cov}(g_1, \pi)^2} \\ &= \beta_1 \frac{\text{Var}(\pi)\text{Var}(g_1) - \text{Cov}(g_1, \pi)^2}{\text{Var}(\pi)\text{Var}(g_1) - \text{Cov}(g_1, \pi)^2} \\ &= \beta_1, \end{aligned} \tag{E.3}$$

and at the unlinked neutral variant (position $j = 2$), we have

$$\begin{aligned} E[\hat{\beta}_2] &= \beta_1 \frac{\text{Var}(\pi)\text{Cov}(g_1, g_2) - \text{Cov}(g_1, \pi)\text{Cov}(g_2, \pi)}{\text{Var}(\pi)\text{Var}(g_2) - \text{Cov}(g_2, \pi)^2} \\ &= 0. \end{aligned} \tag{E.4}$$

E.2.3 Principal component adjusted model

Last, we consider a model that adjusts for two principal components, $\mathbf{u}_1, \mathbf{u}_2$, supposing that the first PC captures global ancestry (i.e., $u_{i1} = \pi_i \; \forall i$) and the second captures some other feature quantified by a random variable $\mathbf{z}$ (i.e., $u_{i2} = z_i \; \forall i$). The expected effect size estimate

at variant j is:

$$E[\hat{\beta}_j] = \beta_1 \frac{V_z(V_\pi C_{g_1,g_j} - C_{g_1,\pi}C_{g_j,\pi}) - V_\pi C_{g_1,z}C_{g_j,z} + C_{\pi,z}(C_{g_1,\pi}C_{g_j,z} + C_{g_1,z}C_{g_j,\pi} - C_{g_1,g}C_{\pi,z})}{V_z(V_\pi V_{g_j} - C^2_{g_j,\pi}) - V_\pi C^2_{g_j,z} + C_{\pi,z}(2C_{g_j,\pi}C_{g_j,z} - V_{g_j}C_{\pi,z})},$$

where $V_a = \widehat{\mathrm{Var}}(a)$ and $C_{a,b} = \widehat{\mathrm{Cov}}(a, b)$.

Proof. Again, this proof follows from similar arguments to that for the unadjusted model, now replacing the design matrix $\mathbf{X}$ with $\begin{pmatrix} \mathbf{1} & \mathbf{g}_j & \boldsymbol{\pi} & \mathbf{z} \end{pmatrix}$. After making this substitution, the rest follows. □

Making the same assumptions as above, we first simplify these results considering a general form of $\mathbf{z}$. At the causal variant ($j = 1$),

$$\begin{aligned} E[\hat{\beta}_1] &= \beta_1 \frac{V_z(V_\pi C_{g_1,g_1} - C_{g_1,\pi}C_{g_1,\pi}) - V_\pi C_{g_1,z}C_{g_1,z} + C_{\pi,z}(C_{g_1,\pi}C_{g_1,z} + C_{g_1,z}C_{g_1,\pi} - C_{g_1,g_1}C_{\pi,z})}{V_z(V_\pi V_{g_1} - C^2_{g_1,\pi}) - V_\pi C^2_{g_1,z} + C_{\pi,z}(2C_{g_1,\pi}C_{g_1,z} - V_{g_1}C_{\pi,z})} \\ &= \beta_1 \frac{V_z(V_\pi V_{g_1} - C^2_{g_1,\pi}) - V_\pi C^2_{g_1,z} + C_{\pi,z}(2C_{g_1,\pi}C_{g_1,z} - V_{g_1}C_{\pi,z})}{V_z(V_\pi V_{g_1} - C^2_{g_1,\pi}) - V_\pi C^2_{g_1,z} + C_{\pi,z}(2C_{g_1,\pi}C_{g_1,z} - V_{g_1}C_{\pi,z})} \\ &= \beta_1, \end{aligned} \tag{E.5}$$

and at the unlinked neutral variant (position $j = 2$),

$$\begin{aligned} E[\hat{\beta}_2] &= \beta_1 \frac{V_z(V_\pi C_{g_1,g_2} - C_{g_1,\pi}C_{g_2,\pi}) - V_\pi C_{g_1,z}C_{g_2,z} + C_{\pi,z}(C_{g_1,\pi}C_{g_2,z} + C_{g_1,z}C_{g_2,\pi} - C_{g_1,g_2}C_{\pi,z})}{V_z(V_\pi V_{g_2} - C^2_{g_2,\pi}) - V_\pi C^2_{g_2,z} + C_{\pi,z}(2C_{g_2,\pi}C_{g_2,z} - V_{g_2}C_{\pi,z})} \\ &= \beta_1 \frac{-V_\pi E[\mathrm{Cov}(g_1, z \mid \pi)]E[\mathrm{Cov}(g_2, z \mid \pi)]}{V_z(V_\pi V_{g_2} - C^2_{g_2,\pi}) - V_\pi C^2_{g_2,z} + C_{\pi,z}(2C_{g_2,\pi}C_{g_2,z} - V_{g_2}C_{\pi,z})}. \end{aligned} \tag{E.6}$$

Now, suppose we make an additional assumption about the form of the second principal component. In particular, assume that $\mathbf{z} = \mathbf{z}_g = z_1\mathbf{g}_1 + z_2\mathbf{g}_2 + \mathbf{e}$, $\mathbf{e} \sim (\mu_e, \sigma^2_e)$ for some scalars z_1, z_2. In other words, the 2nd PC captures genotypes at two variants, one of which is the causal variant ($j = 1$) and the other is an unlinked neutral variant ($j = 2$). Then, at

the causal variant, the expected effect size estimate remains

$$E[\hat{\beta}_1] = \beta_1, \tag{E.7}$$

and at the unlinked neutral variant, we now have

$$\begin{aligned} E[\hat{\beta}_2] = \beta_1 & \frac{-4z_1 z_2 V_\pi}{V_z(V_\pi V_{x_2} - C^2_{x_2,\pi}) - V_\pi C^2_{x_2,z} + C_{\pi,z}(2C_{x_2,\pi}C_{x_2,z} - V_{x_2}C_{\pi,z})} \\ & \times \prod_{j=1}^{2}[p_{j2}(1-p_{j2}) + (p_{j1}-p_{j2})(1-p_{j1}-p_{j2})E_\pi + (p_{j1}-p_{j2})^2(E_\pi - E_\pi^2 - V_\pi)]. \end{aligned} \tag{E.8}$$

Here we can see more directly that the magnitude of the bias at the unlinked neutral variant ($j = 2$) depends on the effect size of the causal variant (β_1), the variability of admixture proportions in the population (V_π), and the strength of the contribution of the causal variant and neutral variant to the principal component (z_1 and z_2, respectively).

E.3 Simulations validating theory

To support these theoretical results, we performed a small simulation study. We generated data according to the data generating mechanism described in Section E.1, and then we compared the observed effect size estimates from GWAS models fit to these simulated data to the expected effect size estimates derived in Section E.2.

We considered a variety of simulation settings, but present results from just a single setting here. Admixture proportions for $n = 5000$ individuals were generated from the distribution $F = \text{Beta}(7, 2)$ (see Fig E.1), allele frequencies at the causal variant were 0.7 (ancestral population 1) and 0.2 (ancestral population 2), allele frequencies at the neutral variant were 0.7 (ancestral population 1) and 0.2 (ancestral population 2), admixture proportions did not have a direct effect on the trait ($\beta_\pi = 0$), and the 2nd PC was generated according to $z_i = z_1 g_{i1} + z_2 g_{i2} + e_i$ for a range of values z_1, z_2 and added noise $e_i \overset{iid}{\sim} N(0, 0.25^2)$.

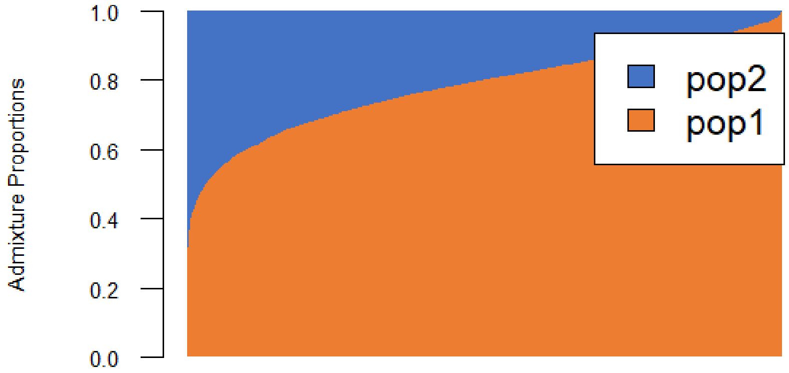

Fig E.1. Barplot of simulated admixture proportions.

In Figs E.2, E.3, and E.4 we plot the effect size estimates from GWAS models that we observe in our simulation study, and compare these observed effect size estimates to the expected effect sizes based on our analytic results (Section E.2), as well as the true effect sizes based on the data-generating mechanism (Section E.1). For all three types of models, we see a perfect correspondence between the observed effect sizes and the expected effect sizes provided in Section E.2, validating our theoretical derivations.

Comparing the observed and expected effect sizes to the true effect sizes provides insight into the magnitude of bias that can be expected from each model. Fig E.2 presents results using the unadjusted GWAS model. We see a departure between the true effect size (0) and the observed and expected effect sizes at the unlinked neutral variant (SNP 2), confirming that models that fail to adjust for ancestral heterogeneity can yield biased estimates of the effect size even when global ancestry does not have a direct effect on the trait ($\beta_\pi = 0$).

In Fig E.3, we consider the case of a GWAS model that adjusts for ancestral heterogeneity by including the true admixture proportions as a covariate. In this case, we see a perfect correspondence between the observed, expected, and true effect sizes for both the causal and unlinked neutral variants. This confirms, as our theory suggests, that models that appropriately adjust for ancestral heterogeneity will yield unbiased estimates of variant effect sizes (i.e., $E[\hat{\beta}_j] = \beta_j$).

Fig E.4 illustrates the impact of adjusting for a principal component that captures local

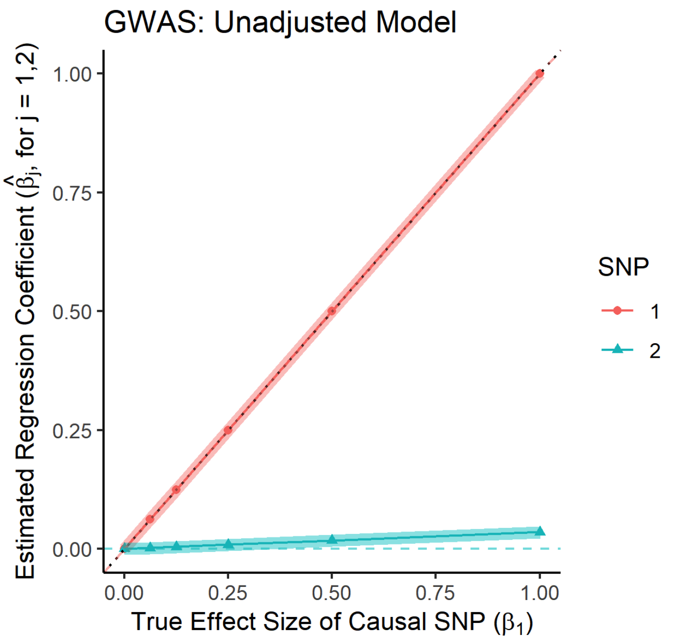

Fig E.2. Comparison of observed, expected, and true effect sizes from unadjusted GWAS models applied to simulated data.
Observed effect sizes are represented by the thin solid lines with points (red with dots = SNP 1, blue with triangles = SNP 2). Expected effect sizes from Section E.2 are represented by the wider and faintly colored solid lines (red = SNP 1, blue = SNP 2). True effect sizes are represented by the dashed lines (red = SNP 1, blue = SNP 2). The $y = x$ line is also provided for reference (dotted black line).

genomic features (in this case, the genotype of variants 1 and 2) instead of global ancestry. Specifically, the second PC was generated according to the equation $z_1 g_{i1} + z_2 g_{i2} + N(0, 0.25^2)$ for scalars z_1, z_2. Although the effect sizes estimates for variant 1 are unbiased across all simulation settings, confirming our theoretical result that $E[\hat{\beta}_1] = \beta_1$ (see Section E.2), we see that the observed and expected effect sizes for SNP 2 often deviate from the truth. The only situations in which effect size estimates for this neutral unlinked variant are unbiased (i.e., $E[\hat{\beta}_2] = \beta_2 = 0$) are when the PC is not actually affected by the causal variant (i.e., $z_1 = 0$) or when the PC is not affected by the neutral variant (i.e., $z_2 = 0$). In other words, as long as the extraneous PC captures the genotypes of both the causal variant and the unlinked neutral variant, we see that effect sizes are biased away from zero at this neutral variant. The magnitude of this bias increases with the strength of the relationship between the variants and the PC (i.e., as we increase z_1 and/or z_2).

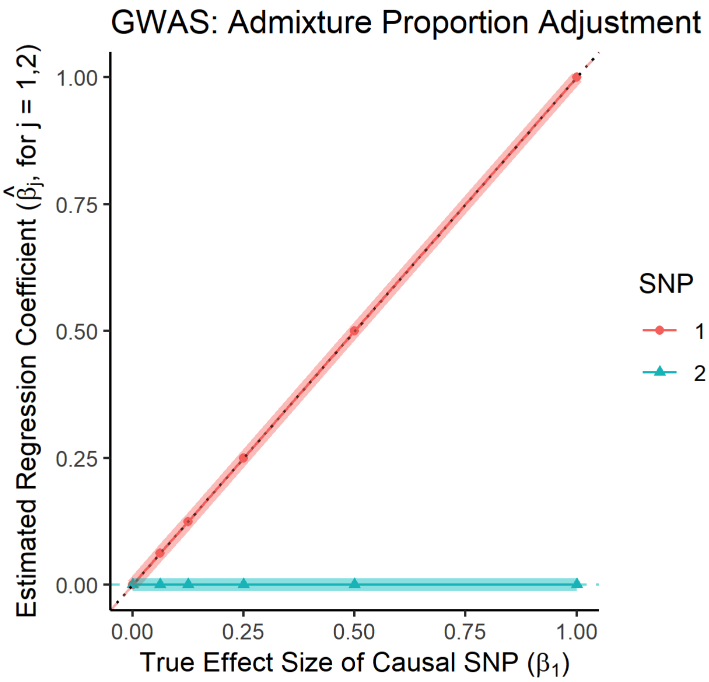

Fig E.3. Comparison of observed, expected, and true effect sizes from GWAS models adjusting for admixture proportions in simulated data.
Observed effect sizes are represented by the thin solid lines with points (red with dots = SNP 1, blue with triangles = SNP 2). Expected effect sizes from Section E.2 are represented by the wider and faintly colored solid lines (red = SNP 1, blue = SNP 2). True effect sizes are represented by the dashed lines (red = SNP 1, blue = SNP 2). The $y = x$ line is also provided for reference (dotted black line).

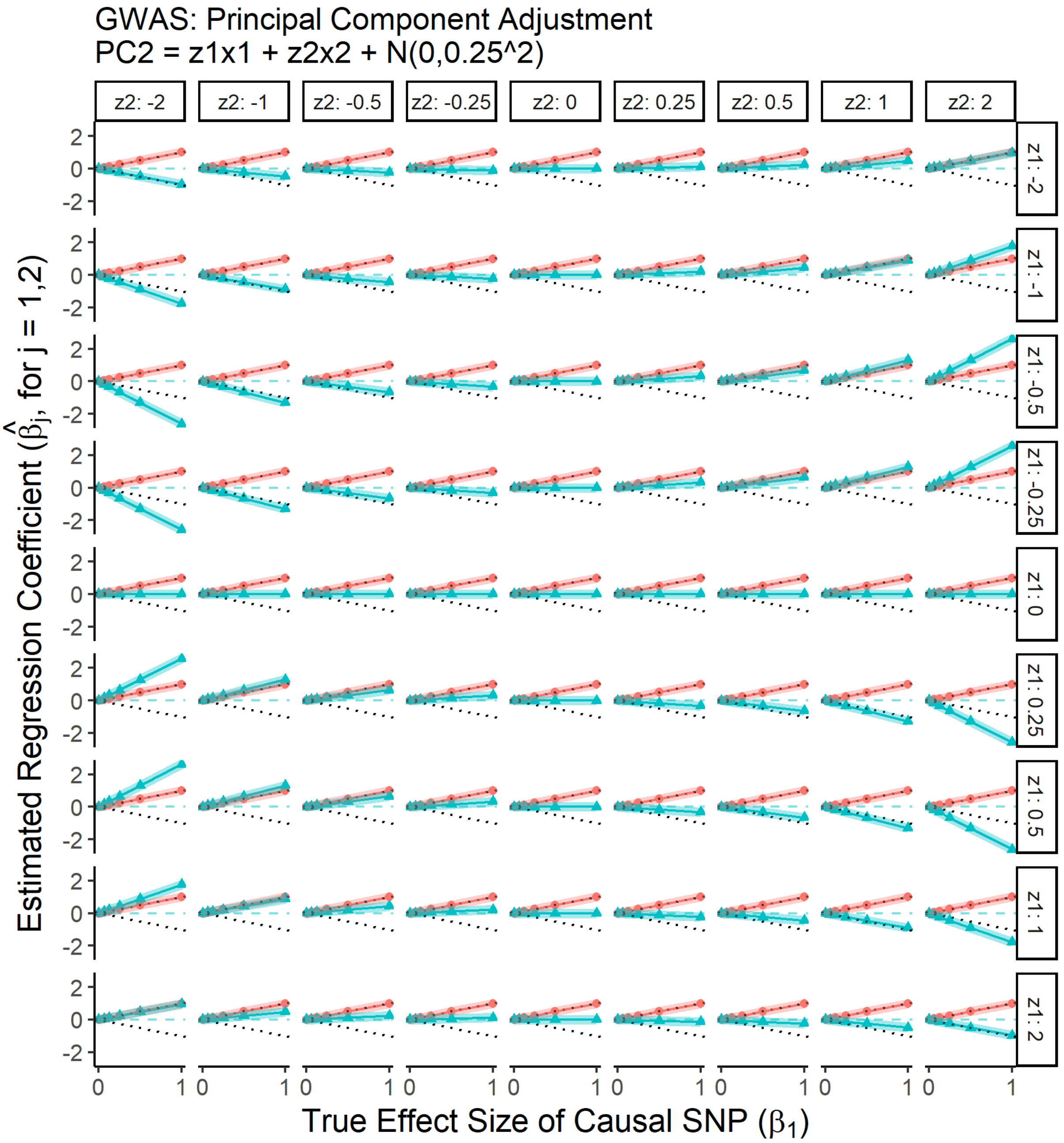

Fig E.4. Comparison of observed, expected, and true effect sizes from GWAS models adjusting for two principal components.

The first PC captures global ancestry, but the second PC was generated according to the equation $z_1 g_{i1} + z_2 g_{i2} + N(0, 0.25^2)$, changing the scalars z_1, z_2 in each panel. Observed effect sizes are represented by the thin solid lines with points (red with dots = SNP 1, blue with triangles = SNP 2). Expected effect sizes are represented by the wider and faintly colored solid lines (red = SNP 1, blue = SNP 2). True effect sizes are represented by the dashed lines (red = SNP 1, blue = SNP 2). The $y = x$ line is also provided for reference (dotted black line).

F Spurious Association Simulation Studies

We performed two simulation studies, one using data from WHI SHARe and the other using data from TOPMed, to support and expand upon the findings from our GWAS effect size derivations (see Section E). These simulations are described in the main paper, but we provide additional results below.

F.1 WHI SHARe

Briefly, we simulated traits influenced by a single causal variant, changing the location of that causal variant in different replicates of the simulation study, and then compared the number of spurious associations observed when GWAS models adjust for ancestral heterogeneity in different ways. Sections 2.5 and 2.6 present the main results of this simulation study.

In the main paper, Fig 5 shows the average number of spurious associations observed for different choices of GWAS models. Here, we supplement these results with additional choices of the effect size of the causal variant, as well as a comparison to a model that makes no adjustment for ancestral heterogeneity whatsoever. See Fig F.1 for these expanded results. As in Fig 5, we see a pattern of increased spurious associations as the effect size of the causal variant increases for models adjusting for PCs that capture local genomic features (i.e., the models adjusting for 4 PCs, without prior LD pruning). We also now see the average number of spurious associations for models that do not make any adjustment for ancestral heterogeneity (i.e., the pink lines in Fig F.1). Even though global ancestry does not have a direct effect on the trait in this simulation study (i.e,. $\beta_\pi = 0$), we still see considerably elevated rates of spurious associations for this unadjusted model — unless the causal variant has similar allele frequencies in the two ancestral populations (Fig F.1B). In settings where the difference in ancestral allele frequencies is larger (panels C, D, and E of Fig F.1), the average number of spurious associations quickly rises as the effect size of the causal variant increases.

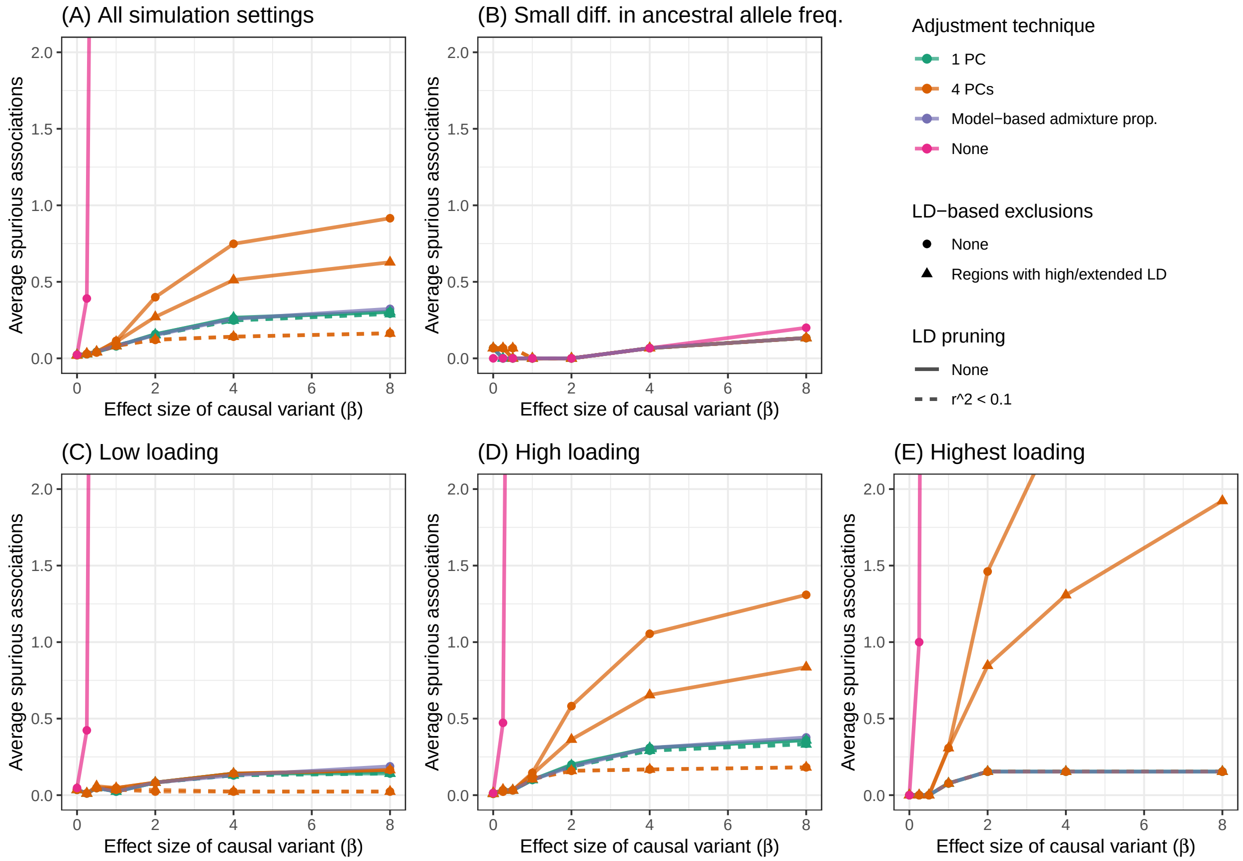

Fig F.1. Comparison of the number of spurious associations in genome-wide association studies in WHI SHARe African Americans using different approaches to adjust for ancestral heterogeneity.

Panel (A) displays the average number of spurious associations that were observed across all simulation settings. Remaining panels focus on the subset of simulation settings in which the causal variant has (B) a small difference in ancestral allele frequencies, (C) low SNP loadings for each of the first four PCs, (D) a high SNP loading for at least one of the first four PCs, or (E) the highest SNP loading on its chromosome for one of the first four PCs. Within each panel, we compare the number of spurious associations when GWAS models adjust for estimated admixture proportions, 1 PC (with or without LD pruning and/or Table 1 exclusions), or 4 PCs (with or without LD pruning and/or Table 1 exclusions). Results shown here are for simulated traits with a single causal variant with effect size (β) ranging from 0 to 8.

As we see in Figs F.1 and 5, LD pruning proves more effective than excluding known high-LD regions alone. This is true, on average, across all simulation settings and also true in the case of the specific scenario presented in Fig 4. Panel F of that figure presents a Manhattan plot from GWAS adjusting for four PCs when *both* pruning and exclusions were performed. Here, we also present results when these pre-processing steps were performed individually (Fig F.2). We see that LD pruning alone is effective in preventing the spurious association on chromosome 6 (Fig F.2A), but exclusions alone is not (Fig F.2B).

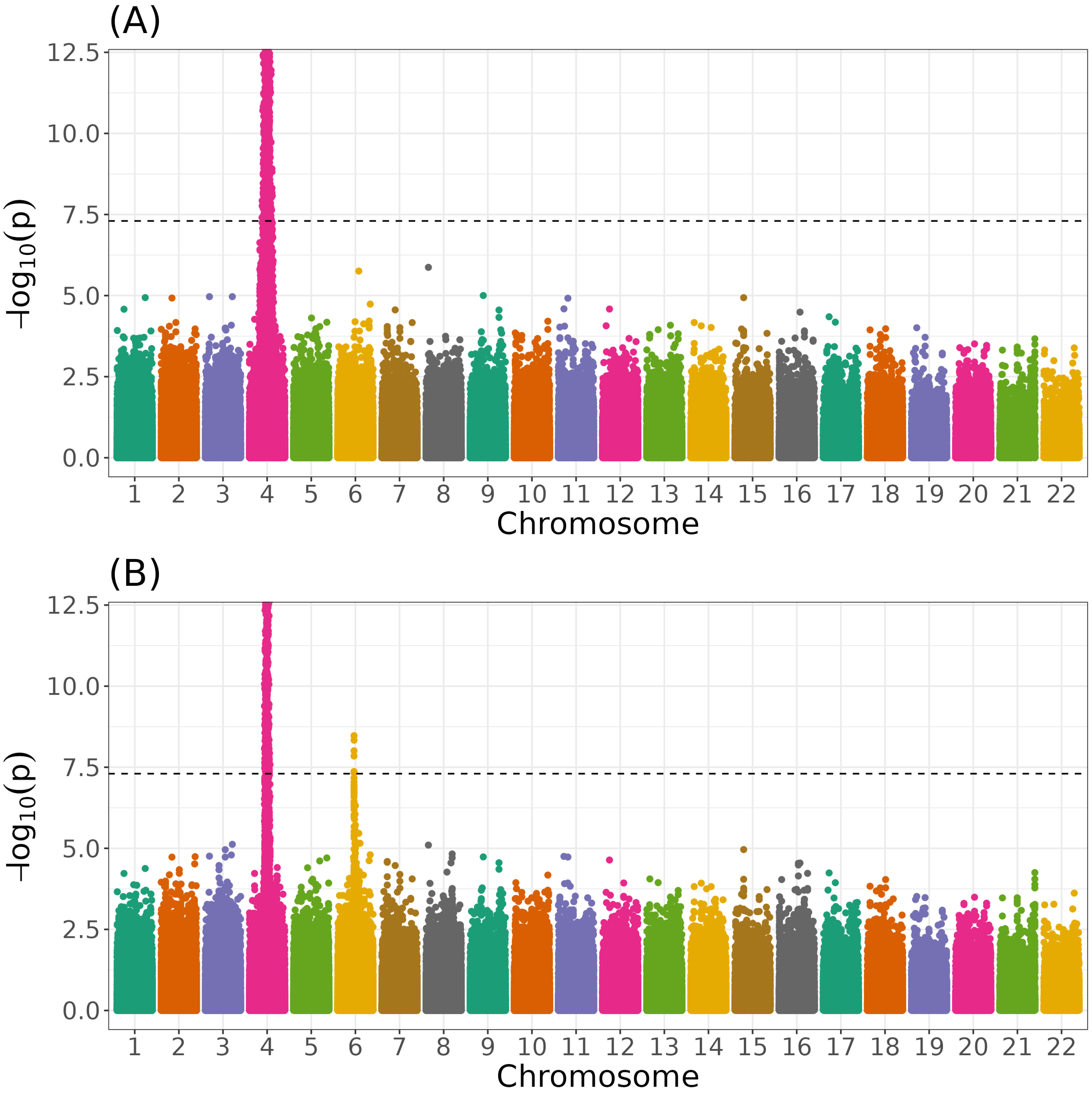

Fig F.2. Manhattan plots from GWAS in WHI SHARe adjusting for 4 PCs, where PCs were generated after either LD pruning or Table 1 exclusions, but not both.
In this example, the simulated trait depends only on the genotype at a single variant on chromosome 4 (as in Fig 4). Panels present results using different adjustment approaches: (A) four PCs, with PCs calculated after LD pruning ($r^2 < 0.1$, window size = 0.5 Mb) and (B) four PCs, with PCs calculated after Table 1 exclusions. The horizontal dashed line in all panels represents the genome-wide significance threshold of 5×10^{-8}. Note that we have restricted the range of the y-axis to [0, 12.5] to facilitate comparisons across panels and focus attention near the 5×10^{-8} threshold. There are some SNPs (exclusively located on chromosome 4, in the case of panels B–F) with small p-values that extend beyond the range of the plot.

F.2 TOPMed

We also performed simulations using whole genome sequence data from the Trans-Omics for Precision Medicine Project, with the goal of illustrating what happens when models adjust for extra PCs that capture multiple local genomic features instead of global ancestry. For each individual, we simulated a quantitative trait that depended only on their genotype at a single variant. We also constructed two "fake" PCs. The first PC was set equal to the estimated African admixture proportion. The second PC was generated such that it depended on the genotype at this same causal variant, as well as a second variant on another chromosome. Here, we focus on an example where the causal variant is located on chromosome 8 and the second variant contributing to the "extra" PC is located on chromosome 6. Fig F.3 shows that we see similar patterns in the correlation between this PC and genotypes as we did with real PCs in WHI SHARe, TOPMed JHS, and TOPMed COPDGene African Americans. These PC-genotype correlation plots clearly show—as we know to be true, by design—that the second PC is driven by variants on two chromosomes (6 and 8) rather than detecting genome-wide ancestry.

We then investigated the impact of including this extraneous PC in GWAS models. Our results again mirror the patterns observed in our WHI SHARe simulations. Fig F.4 presents Manhattan plots from a single simulation replicate, comparing results from a model that adjusted for just the first principal component (top panel) versus a model that adjusted for both PCs (bottom panel). In both cases, we see a genome-wide significant association on chromosome 8 — the location of the true causal variant. However, in the case of the model adjusting for two PCs, we also see a spurious association on chromosome 6 — the location of the second variant that contributes to the second principal component. The second PC plays the role of a collider variable in this setting, and adjusting for it has induced a spurious association. It is worth noting that the quantile-quantile (QQ) plots and inflation factors for these two analyses are indistinguishable (Fig F.5), so those tools alone are not sufficient for detecting this issue.

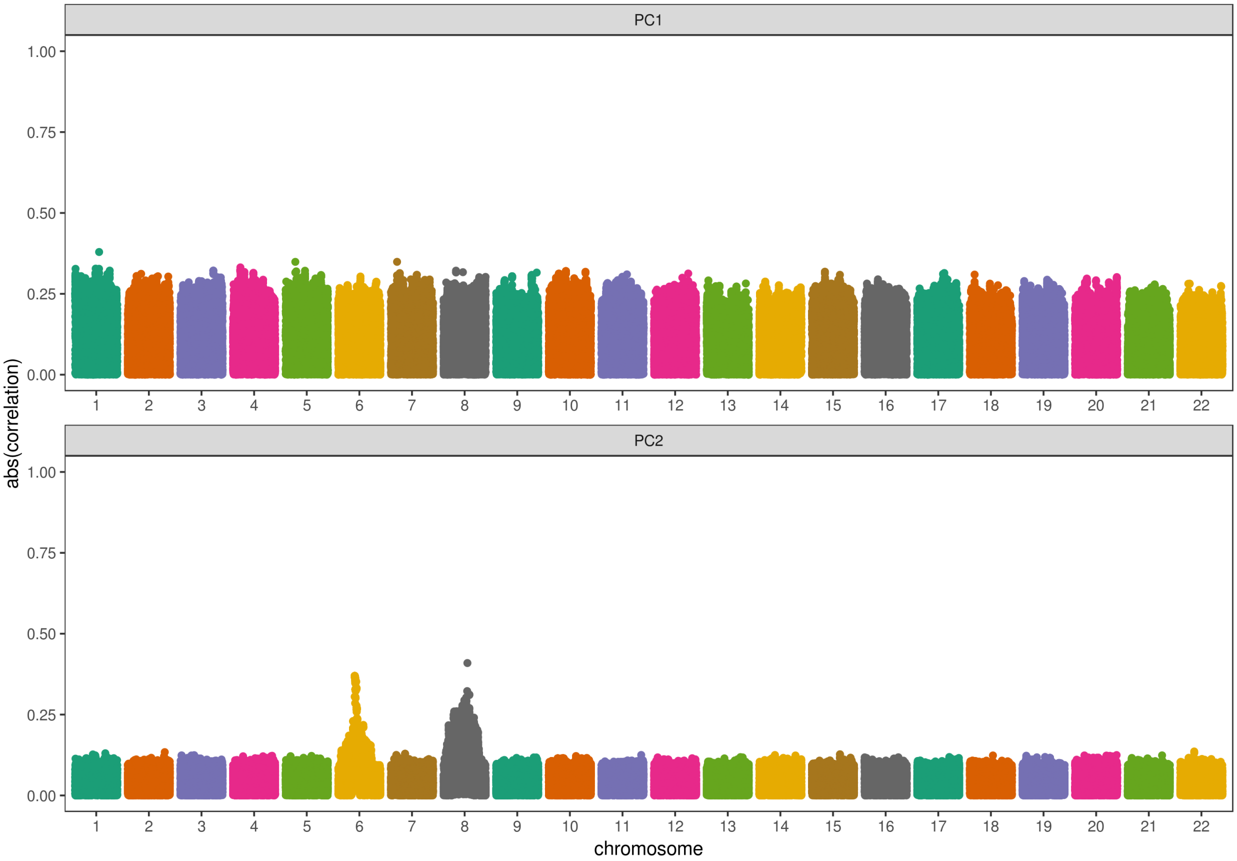

Fig F.3. Correlation between fake PCs (i.e., PCs that were constructed such that the first captures genetic ancestry but the second captures genotype at two variants on chromosomes 6 and 8) in TOPMed JHS African Americans.
Each panel plots the absolute value of the correlation between principal components and genotypes (on the y-axis) versus the position along the genome (x-axis). Panels are organized vertically according to which PC is being investigated (1, 2). Peaks in this plot indicate that a variant has a larger *loading*, i.e., a larger contribution to that principal component.

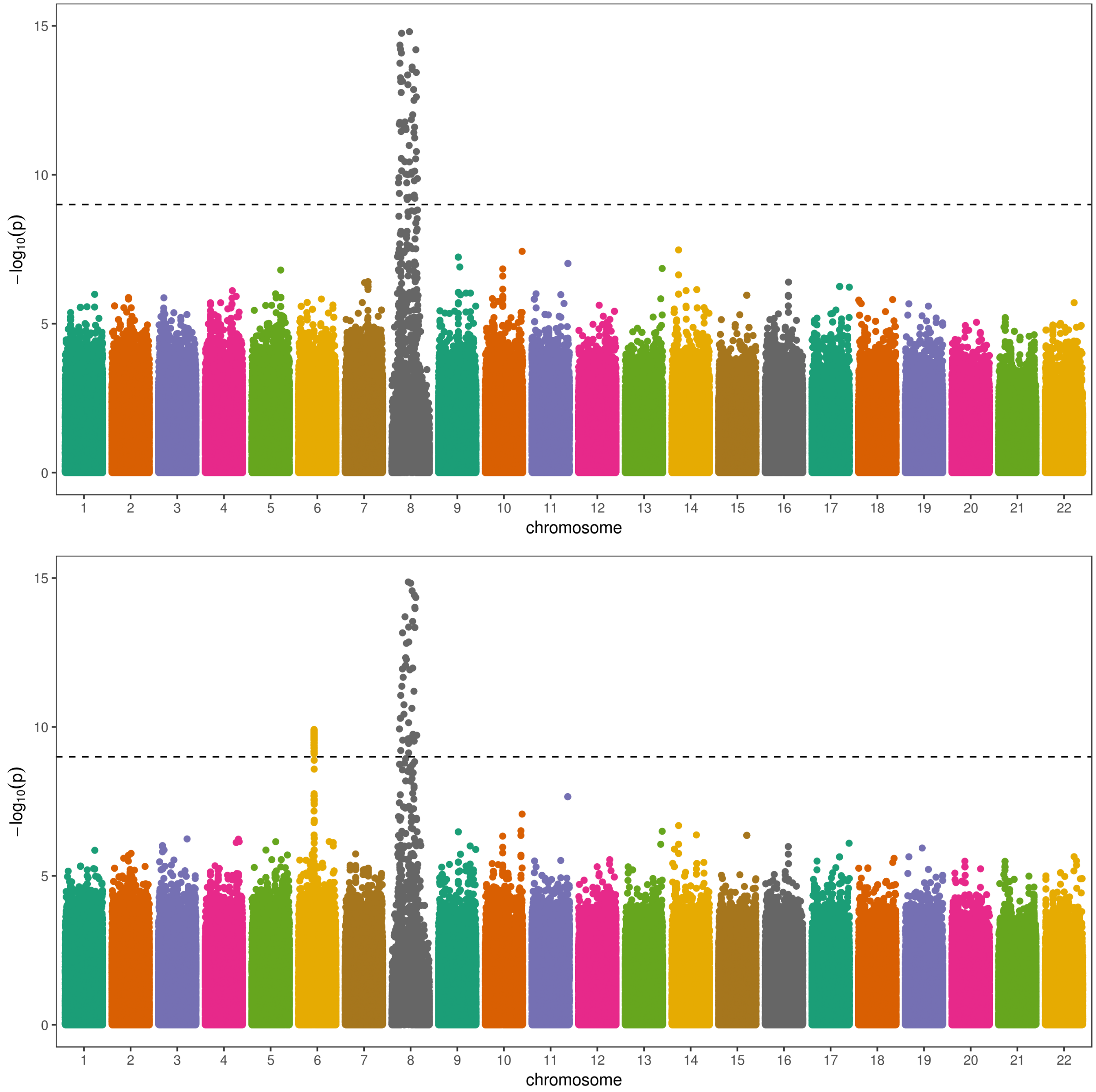

Fig F.4. Manhattan plots for GWAS models adjusting for fake PCs (i.e., PCs that were constructed such that the first captures genetic ancestry but the second captures genotype at two variants on chromosomes 6 and 8) in TOPMed JHS African Americans.
The top panel presents results from a model adjusting for only the first PC and the bottom panel presents results from a model adjusting for two PCs. In this simulation setting, there is only one causal variant, located on chromosome 8.

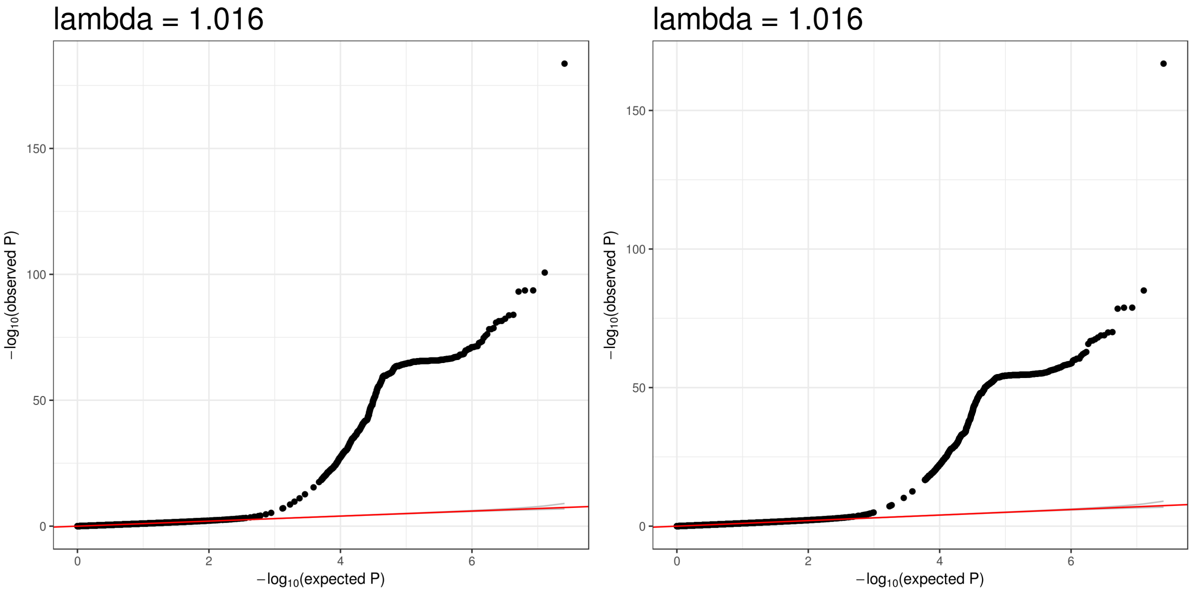

Fig F.5. Quantile-quantile (QQ) plots and inflation factors (λ) for GWAS models adjusting for fake PCs (i.e., PCs that were constructed such that the first captures genetic ancestry but the second captures genotype at two variants on chromosomes 6 and 8) in TOPMed JHS African Americans.

The left panel presents results from a model adjusting for only the first PC and the right panel presents results from a model adjusting for two PCs. The two plots are indistinguishable, and the inflation factors are identical, despite the fact that the model adjusting for two PCs (right panel) resulted in a spurious association.

G List of WHI Investigators

The authors wish to acknowledge the many investigators who have contributed to the Women's Health Initiative (WHI). In particular:

- **Program Office**: (National Heart, Lung, and Blood Institute, Bethesda, Maryland) Jacques Rossouw, Shari Ludlam, Joan McGowan, Leslie Ford, and Nancy Geller
- **Clinical Coordinating Center**: (Fred Hutchinson Cancer Research Center, Seattle, WA) Garnet Anderson, Ross Prentice, Andrea LaCroix, and Charles Kooperberg
- **Investigators and Academic Centers**: (Brigham and Women's Hospital, Harvard Medical School, Boston, MA) JoAnn E. Manson; (MedStar Health Research Institute/Howard University, Washington, DC) Barbara V. Howard; (Stanford Prevention Research Center, Stanford, CA) Marcia L. Stefanick; (The Ohio State University, Columbus, OH) Rebecca Jackson; (University of Arizona, Tucson/Phoenix, AZ) Cynthia A. Thomson; (University at Buffalo, Buffalo, NY) Jean Wactawski-Wende; (University of Florida, Gainesville/Jacksonville, FL) Marian Limacher; (University of Iowa, Iowa City/Davenport, IA) Jennifer Robinson; (University of Pittsburgh, Pittsburgh, PA) Lewis Kuller; (Wake Forest University School of Medicine, Winston-Salem, NC) Sally Shumaker; (University of Nevada, Reno, NV) Robert Brunner
- **Women's Health Initiative Memory Study**: (Wake Forest University School of Medicine, Winston-Salem, NC) Mark Espeland

A full list of the investigators who have contributed to WHI can be found here: https://s3-us-west-2.amazonaws.com/www-whi-org/wp-content/uploads/WHI-Investigator-Long-List.pdf

Supplemental References

[1] Privé F, Aschard H, Ziyatdinov A, Blum MG. Efficient analysis of large-scale genome-wide data with two R packages: bigstatsr and bigsnpr. Bioinformatics. 2018;34(16):2781–2787.

[2] Weale ME. In: Barnes MR, Breen G, editors. Quality Control for Genome-Wide Association Studies. Totowa, NJ: Humana Press; 2010. p. 341–372. Available from: https://doi.org/10.1007/978-1-60327-367-1_19.

[3] Galinsky KJ, Bhatia G, Loh PR, Georgiev S, Mukherjee S, Patterson NJ, et al. Fast principal-component analysis reveals convergent evolution of ADH1B in Europe and East Asia. The American Journal of Human Genetics. 2016;98(3):456–472.

[4] Zou F, Lee S, Knowles MR, Wright FA. Quantification of population structure using correlated SNPs by shrinkage principal components. Human Heredity. 2010;70(1):9–22.

[5] Fellay J, Shianna KV, Ge D, Colombo S, Ledergerber B, Weale M, et al. A whole-genome association study of major determinants for host control of HIV-1. Science. 2007;317(5840):944–947.

[6] Reed E, Nunez S, Kulp D, Qian J, Reilly MP, Foulkes AS. A guide to genome-wide association analysis and post-analytic interrogation. Statistics in Medicine. 2015;34(28):3769–3792.

[7] Novembre J, Johnson T, Bryc K, Kutalik Z, Boyko AR, Auton A, et al. Genes mirror geography within Europe. Nature. 2008;456(7218):98–101.

[8] Anderson CA, Pettersson FH, Clarke GM, Cardon LR, Morris AP, Zondervan KT. Data quality control in genetic case-control association studies. Nature Protocols. 2010;5(9):1564–1573.

[9] Zheng X, Levine D, Shen J, Gogarten SM, Laurie C, Weir BS. A high-performance computing toolset for relatedness and principal component analysis of SNP data. Bioinformatics. 2012;28(24):3326–3328.

[10] Conomos MP, Laurie CA, Stilp AM, Gogarten SM, McHugh CP, Nelson SC, et al. Genetic diversity and association studies in US Hispanic/Latino populations: applications in the Hispanic Community Health Study/Study of Latinos. The American Journal of Human Genetics. 2016;98(1):165–184.

[11] Privé F, Luu K, Blum MG, McGrath JJ, Vilhjálmsson BJ. Efficient toolkit implementing best practices for principal component analysis of population genetic data. Bioinformatics. 2020;36(16):4449–4457.

[12] Maples BK, Gravel S, Kenny EE, Bustamante CD. RFMix: a discriminative modeling approach for rapid and robust local-ancestry inference. The American Journal of Human Genetics. 2013;93(2):278–288.

[13] Alexander DH, Novembre J, Lange K. Fast model-based estimation of ancestry in unrelated individuals. Genome Research. 2009;19(9):1655–1664.
